# Genome of Kumamoto Oyster *Crassostrea sikamea* Provides Insights Into Bivalve Evolution and Environmental Adaptation

**DOI:** 10.1111/eva.70100

**Published:** 2025-04-24

**Authors:** Sheng Liu, Youli Liu, Ximing Guo, Naoki Itoh, Guangqiu Chang, Zhihua Lin, Qinggang Xue

**Affiliations:** ^1^ Institute of Mariculture Breeding and Seed Industry Zhejiang Wanli University Ninghai Zhejiang China; ^2^ Zhejiang Key Laboratory of Aquatic Germplasm Resource Zhejiang Wanli University Ningbo Zhejiang China; ^3^ Haskin Shellfish Research Laboratory Rutgers University Port Norris New Jersey USA; ^4^ Laboratory of Fish Diseases, Graduate School of Agricultural and Life Sciences The University of Tokyo Bunkyo Tokyo Japan

**Keywords:** bivalve mollusc, chromosomal rearrangement, domestication, environmental adaptation, karyotype evolution, oyster aquaculture

## Abstract

The Kumamoto oyster, *Crassostrea sikamea*, is a marine bivalve naturally distributed along the coasts of southern China and southern Japan, with a hatchery population that has been under domestication in the United States since its introduction from Japan in the 1940s. To understand its evolutionary history and environmental adaptation, we produced a chromosome‐level genome assembly of *C. sikamea* and conducted whole‐genome resequencing of 141 individuals from the US hatchery population and six wild populations from China and Japan. The assembled genome of *C. sikamea* has a size of 616 Mb covering all 10 chromosomes with a contig N50 of 4.21 Mb and a scaffold N50 of 62.25 Mb. Phylogenetic analysis indicated that *C. sikamea* diverged from the *Crassostrea angulata* and *Crassostrea gigas* clade about 9.9 million years ago. Synteny analysis revealed significant chromosomal rearrangements during bivalve evolution leading to oysters, but remarkable conservation of all 10 oyster chromosomes over ~180 million years, a surprising disparity in chromosomal evolution. Phylogenetic analysis produced three distinct clusters for the US, Japanese, and Chinese populations, with the US population closer to the Japanese population, confirming its origin. No differentiation was detected among the five Chinese populations, indicating strong gene flow. Between the US and Japan populations, 402 genes exhibited selection signals, including three myosin heavy chain genes that were also differentiated in domesticated lines of the eastern oyster, suggesting changes in these genes may be important for domestic production. Among the 768 genes showing selection signals between natural populations of Japan and China, genes related to stress response are most enriched, suggesting responding to environmental stress is critical for local adaptation. These findings provide insights into bivalve evolution and environmental adaptation, as well as useful resources for comparative genomics and genetic improvement of cultured Kumamoto oyster stocks.

## 1 Introduction

Oysters are widely distributed in world oceans and play important roles in coastal and estuary ecology. They are filter‐feeders and habitat engineers, providing critical ecological services to marine ecosystems (Beck et al. 2011; Grabowski et al. 2012). As sessile species thriving in intertidal zones of estuaries where environmental conditions fluctuate greatly, oysters are resilient and provide good model species for studying environmental adaptation of sessile marine invertebrates. There are about 100 species of living oysters around the world, and many of them are important fishery and aquaculture species (Guo et al. 2018). Oyster aquaculture is an important industry globally, with an annual production of ~7 million tons (FAO 2024). Because of its ecological and economic significance, great efforts have been made to understand the biology, diversity, and evolution of oysters (Bayne 2017; Guo et al. 2018). Genomes of several oyster species have been sequenced, including the Pacific oyster *Crassostrea gigas* (Zhang et al. 2012; Penaloza et al. 2021; Qi et al. 2021), eastern oyster *Crassostrea virginica* (Puritz et al. 2023), Suminoe oyster *Crassostrea ariakensis* (Li, Dai, et al. 2021), Hong Kong oyster *Crassostrea hongkongensis* (Zhang et al. 2022), European flat oyster *Ostrea edulis* (Boutet et al. 2022; Gundappa et al. 2022), and Sydney rock oyster *Saccostrea glomerata* (Powell et al. 2018). The sequencing and analysis of oyster genomes have provided unprecedented insights into oyster biology and evolution, as well as valuable genomic resources for the genetic improvement of cultured stocks.

Kumamoto oyster, *Crassostrea sikamea*, is a common species found along the coasts of East Asia, ranging from southern Japan, southern China to Vietnam (Banks et al. 1994; Hamaguchi et al. 2013; Wang et al. 2013; In et al. 2017). Kumamoto oyster is a relatively slow‐growing species with small but deeply cupped shells compared to other *Crassostrea* oysters. Its small size and unique shells may provide a good case for studying growth regulation. Kumamoto oyster is also a popular aquaculture species favored for its taste and esthetic shell attributes. The species prefers the upper region of the intertidal zone and can tolerate harsher environmental conditions than its sister species. In addition, Kumamoto oysters seem to be more tolerant to *Ostreid Herpesvirus* 1, showing much lower mortality under challenge than Pacific oysters (Friedman et al. 2020). Due to the lack of genomic resources, however, the genomic basis for its slow growth and environmental resilience has not been well studied. Genomic analysis had to rely on related species, such as *C. gigas* (Hu and Dong 2022), but the mapping rate of *C. sikamea* RNA‐seq reads to the *C. gigas* genome was low (45%) and of limited use (Qi et al. 2021).

The use and exchange of genetic resources have played an important role in the development of molluscan aquaculture (Guo 2009). Kumamoto oysters were introduced to the Northwest coast of the United States from the Ariake Sea in Japan about 80 years ago (Sekino 2009). As there were no wild populations in the United States, the introduced *C. sikamea* has been produced in hatcheries and cultured on oyster farms. Hatchery production and aquaculture may produce genetic changes due to intentional (keeping large larvae and seed) or unintentional (adaptation to culturing conditions) selections, both contributing to the domestication/adaptation process. The environment in the Pacific Northwest of the United States, which has a narrow variation of ocean temperature (~8°C–19°C) (Heare et al. 2017), differs significantly from *C. sikamea*'s native environment in Asia, where temperature may vary in a range of 5°C–30°C (Li, Dai, et al. 2021). The American population of *C. sikamea* introduced from Japan has shown signs of domestication, such as fast growth and much larger individual size (Ma et al. 2022), as well as decreased effective population size (Hedgecock et al. 1993). Although oyster farming began over 2000 years ago (Guo et al. 1999), oyster breeding has a relatively short history with the oldest breeding program dating to only 1960 (Guo 2021). Even a short period of domestication may lead to genetic differentiation at the genome level, which is not well understood (Zhao et al. 2024). The introduced American population of *C. sikamea* provides a unique opportunity to study the effects of domestication and/or environmental adaptation. For *C. sikamea* in this study, we consider domestication to be part of environmental adaptation, as we cannot separate the effects of hatchery and farming practices from environmental adaptation for the American population.

Genome sequencing and resequencing of individuals from diverse populations will greatly enhance our understanding of oyster population genetics (Hu and Dong 2022). Additionally, it may provide insights into the genetic mechanisms that enable *C. sikamea* to thrive in more challenging environmental conditions compared to its sister species. More importantly, these genomic resources can facilitate efficient genotyping, which is essential for genomic studies and selective breeding programs. They can also support the development of single‐nucleotide polymorphism (SNP) arrays. These SNP arrays can advance genomic research by enabling more precise and comprehensive analyses, such as genome‐wide association studies for the identification of genes for production traits. Ultimately, this will accelerate the genetic improvement of the Kumamoto oyster by allowing genomic selection for important traits such as growth and disease resistance (Guo et al. 2023; Zhao et al. 2024; Wang et al. 2025).

In this study, we sequenced and analyzed the genome of *C. sikamea* to gain insights into its biology, evolutionary history, environmental adaptation, and possible changes from domestication. We combined long‐read sequencing and two long‐range scaffolding techniques to obtain a high‐quality chromosome‐level genome assembly for *C. sikamea*. We conducted synteny analysis to better understand the karyotypic evolution of bivalves leading to oysters. We resequenced wild oysters from China and Japan and hatchery populations from the United States and identified genomic regions possibly affected by domestication and environmental adaptation. Our findings provide insights into bivalve evolution and environmental adaptation, as well as useful resources for genetic improvement of cultured Kumamoto oyster stocks.

## 2 Materials and Methods

### 2.1 Animals and Sampling

Kumamoto oysters, *C. sikamea*, were collected from oyster farms at Xidian town, Xiangshan bay (29.47° N, 121.42° E) in Zhejiang Province, China. Oyster species were identified according to the PCR product length polymorphism of cytochrome oxidase C subunit I (COI) and internal transcribed spacer 1 (ITS1) (Wang and Guo 2008; Xu et al. 2019). Because of their small size, three unsexed 1.5‐year‐old *C. sikamea* of 25–27 g in whole body weight were randomly sampled, with labial palps from one being used to extract DNA for genome short‐read and long‐read sequencing, mixed tissues from one to extract DNA for high‐throughput chromosome conformation capture (Hi‐C) sequencing, and different organs, including the gill, mantle, muscle, gonad, digestive gland, and labial palps from the other being used to extract total mRNAs separately for the transcriptomic analysis. The sampled tissues were snap frozen in liquid nitrogen after being rinsed in PBS and then used for DNA or mRNA extraction. DNAs were extracted with the Phenol–Chloroform Isoamyl alcohol method. Total RNAs were extracted from tissues using TRIzol Reagent (OMEGA, USA) according to the manufacturer's instruction. The extracted DNA and RNA samples were assessed for purity using a NanoDrop 2000 microspectrophotometer, for concentration using a Qubit fluorometer, and for integrity using an Agilent 4200 Bioanalyzer.

### 2.2 Genome Sequencing and Assembling

#### 2.2.1 Library Construction and Sequencing

For short‐read genome survey sequencing, two paired‐end libraries with 350 bp insert size were constructed using the extracted DNA from labial palps and sequenced with the Novaseq PE150 platform (Illumina), and the data were used for the *k*‐mer analysis.

For long‐read genome sequencing, single‐molecule real‐time (SMRT) circular consensus sequencing libraries were constructed according to Pacific Bioscience instructions. Briefly, a total of 15 μg genomic DNA was sheared to ~15 kb, and sequencing libraries were constructed for HiFi sequencing on the PacBio Sequel II System (Pacific Biosciences of California Inc.). The constructed libraries were sequenced on two SMRT cells.

For chromosomal assembly of the genome, Hi‐C libraries with an insert size of 50 bp were constructed and sequenced with the Novaseq PE150 platform (Illumina). Illumina Novaseq raw reads with adapters or more than 10% missing sequencing or low‐quality reads were filtered out to obtain high‐quality clean reads for subsequent analysis. For data generated from the PacBio Sequel II platform, adapters or low‐quality reads were deleted by SMRTlink v8.0 with default parameters. The genome sequencing was conducted by Annoroad Gene Technology (Beijing) Co. Ltd.

#### 2.2.2 Genome Size Estimation and Genome Assembly

The genome size and heterozygosity were estimated using *k*‐mer analysis, and the number of *k*‐mers was calculated using Jellyfish (Marcais and Kingsford 2011). The genome size was estimated using the formula G = *k*‐mer_number/*k*‐mer depth using the paired‐end Illumina sequence data.

The PacBio long reads were used for contig‐level assembly of the genome by Canu v2.1.1 (Koren et al. 2017) and Hifiasm v0.13‐r308 (Cheng et al. 2021) with default parameters. The primary assembly of the *C. sikamea* genome, with a total length of 1.07 Gb containing 944 contigs and an N50 of 3.15 Mb, was almost twice as large as the expected genome size due to redundancy. A similar phenomenon was observed in assembling the *C. gigas* genome (Penaloza et al. 2021; Qi et al. 2021). Evaluation with BUSCO (v3.0.1, default parameters, http://busco.ezlab.org/) based on eukaryota_odb10 database showed that 755 genes (79.1%) were complete but duplicated in the primary assembly. To remove duplicated contigs, purge_dups (https://github.com/dfguan/purge_dups) was applied to reduce redundancy of the primary assembly to generate a final contig‐level assembly of the genome.

Chromosome‐level genome was assembled with Hi‐C data. Clean reads were first aligned on the genome assembly using the bowtie 2 (v2.2.3; http://bowtie‐bio.sourceforge.net/bowtie2/index.shtml) by setting the parameters as “‐‐very‐sensitive ‐L 20 ‐‐score‐min L, −0.6, −0.2 ‐‐end‐to‐end ‐‐reorder ‐‐rg‐id BMG ‐‐phred33‐quals ‐p 5.” The ligation site of an unmapped read was detected with HiC‐Pro (v2.7.8; https://github.com/nservant/HiC‐Pro) using an exact matching procedure and aligned its 5′‐fraction back onto the genome. The results of both mapping steps were merged into a single alignment file. The LACHESIS (https://github.com/shendurelab/LACHESIS) was used to scale up the primary genome assembly in contigs to chromosome‐scale scaffolds (hereafter pseudochromosomes). To determine the accuracy of the scaling‐up results, the pseudochromosomes predicted by LACHESIS were cut into bins with 100 kb length and heatmap was constructed based on the interaction signals that were revealed by valid mapped read pairs between the bins. The matrix was produced by HiC‐Pro and visualized as a heatmap to show the diagonal patches of strong linkage.

#### 2.2.3 Genome Assembly Assessment

The *C. sikamea* genome assembly was assessed with G + C contents, GC depth, and read map percentage and coverage rate by BWA (Burrows‐Wheeler Aligner, mem ‐M ‐k 30). First, A/T/C/G base content of the final assembly was calculated and compared to assess whether the GC content was normal. GC depth analysis was conducted to check whether the depth coverage was uniform, and the assembled sequence was contaminated with sequences from other species. In addition, consistency assessment was also conducted to evaluate the integrity of the genome by mapping the short DNA reads used for the genome survey to the assembly with BWA (mem ‐M ‐k 30). Percentages of mapped reads and coverage rate were calculated to assess the representativeness of the genome. Finally, quality assessment was conducted using BUSCO (v3.0.1) with default parameters based on mollusca_odb10 database (http://busco.ezlab.org/) for genome assembly and predicted proteins.

### 2.3 Genome Annotation

Genomic annotation was conducted to identify repeat sequences, gene structures and functions, and noncoding RNAs (ncRNAs). Repeat sequences were predicted by comparing sequences with known repeat sequences in the database RepBase (https://www.girinst.org/server/RepBase/index.php) using RepeatMasker and Repeatproteinmask (Tarailo‐Graovac and Chen 2009) and by ab initio prediction using RepeatModeler (http://www.repeatmasker.org/RepeatModeler/). Tandem repeat sequences were identified ab initio using the tandem repeat finder (TRF, https://tandem.bu.edu/trf/trf.html) software.

Genes were predicted by homologous protein searches in the *C. sikamea* genome using blastn (http://blast.ncbi.nlm.nih.gov/Blast.cgi) and Genewise (http://www.ebi.ac.uk/~birney/wise2/) (Birney et al. 2004). Ab initio prediction was based on the genomic sequence statistics, including codon frequency and exon–intron distribution using the software Augustus (http://augustus.gobics.de/) (Stanke et al. 2004), SNAP (https://github.com/KorfLab/SNAP) and GeneMark (http://exon.gatech.edu/GeneMark/) (Ter‐Hovhannisyan et al. 2008). In addition, to assist with genome annotation, total RNA from gill, mantle, muscle, gonad, digestive gland, and labial palp was extracted, six transcriptome libraries were constructed for six organs and sequenced with the Novaseq S2 platform (Illumina). Also, an isoform sequencing (Iso‐Seq) library was generated with mixed RNA from the six organs using a SMARTer PCR cDNA synthesis kit (PacBio) according to the manufacturer's instructions and sequenced with the PacBio Sequel II System (Pacific Biosciences of California Inc.). An RNA‐seq‐based prediction was performed using the software PASA (http://pasa.sourceforge.net/) (Roberts et al. 2011). Predicted gene sets were integrated into a non‐redundant and complete gene set using EVidenceModeler (EVM) (http://evidencemodeler.github.io/) (Haas et al. 2008). Gene functions were predicted by comparing with sequences in databases for protein orthology detection and functional annotation including the Nucleotide Sequence Database (NT) and Non‐redundant Protein Sequence Database (NR) at the National Center for Biotechnology Information (NCBI) (https://www.ncbi.nlm.nih.gov/) and by evolutionary genealogy analysis using Non‐supervised Orthologous Groups (eggNOG) database (http://eggnogdb.embl.de/) (Powell et al. 2012), the Gene Ontology (GO) database (http://geneontology.org/page/go‐database) (Ashburner et al. 2000), and the Kyoto Encyclopedia of Genes and Genomes (KEGG) database (http://www.genome.jp/kegg/) (Kanehisa et al. 2012).

Noncoding RNA annotation in the *C. sikamea* genome involves identifying RNA sequences that were not translatable into proteins, encompassing various types, such as ribosomal RNA (rRNA), transfer RNA (tRNA), small nuclear RNA (snRNA), and microRNA (miRNA). For rRNA, snRNA, and miRNA annotation, genomic sequences were compared with known ncRNA families in the Rfam database (http://rfam.xfam.org/) (Griffiths‐Jones et al. 2005). Specifically, for tRNA sequences, the software tRNAscan‐SE (http://lowelab.ucsc.edu/tRNAscan‐SE/) was employed to analyze the genome sequence and predict tRNA locations.

### 2.4 Phylogenetic Analysis

The genome sequences and annotation files of 15 molluscan species, including 12 bivalves, 2 gastropods, and 1 cephalopod, were downloaded from correspondent sequence databases (Table S1). Gene families were identified in the genomes using OrthoFinder (Emms and Kelly 2019). All single‐copy orthologous genes present in all the 15 species were selected and aligned with MUSCLE v3.8.31 (http://www.drive5.com/muscle/) (Edgar 2004), and a maximum likelihood‐based phylogenetic tree was then constructed with the concatenated coding sequences using PhyML v3.0 with default parameters (Guindon et al. 2010). The divergence time of species was estimated using the method of BRMC in mcmctree v4.0 (burn‐in = 20,000, sample‐frequency = 2) of the PAML software package (Yang 2007). Reference divergence times ( *Octopus bimaculoides* ‐ *Lottia gigantea* : 480.0–559.4 Mya; *Lottia gigantea* ‐ *Aplysia californica* : 489 Mya; *Sinonovacula constricta*‐*Cyclina sinensis*: 101.8–471.4 Mya) from TimeTree website (http://timetree.org/) (Kumar et al. 2022) were used to calibrate divergence times between the studied species on the phylogenetic tree.

### 2.5 Genome Synteny Analysis

Genome synteny analysis was conducted between *C. sikamea* (*n* = 10) and seven other bivalve species including oysters *C. gigas* , *C. virginica* , and *Ostrea edulis* (*n* = 10); pearl oyster *Pinctada fucata*; and mussel *Mytilus coruscus* (*n* = 14), Venus clam *Cyclina sinensis*, and blood clam *Scapharca broughtonii* (*n* = 19). The comparisons were done with MCScanX (Wang et al. 2012) in TBtools software (Chen et al. 2020). Briefly, all protein sequences of target species pairs were compared by blastp (e‐value: 10^−5^, number of hits: 5, number of aligns: 5). One‐step MCScanX was conducted based on the blastp results, and the gene order on the chromosomes (according to the annotation file) was visualized in TBtools. For synteny regions, the maximum gap size was set to 25 genes and a minimum syntenic block required 5 genes.

### 2.6 Genome Resequencing of Natural and Hatchery Populations

A total of 123 wild *C. sikamea* oysters representing 5 geographical populations from the Chinese coasts and 1 population on the Japanese coast, and 18 oysters representing a cultured population in the United States were sampled for genome resequencing (Table S2). The wild oysters were collected in December 2021, and the cultured oysters were purchased from Taylor Shellfish Company in Shelton, Washington, USA in June 2021. Local water temperatures during 2010–2019 in China and Japan were obtained from an online database (Cao et al. 2021). For Dabob Bay where the American population was cultured, data for September 2013–August 2014 were extracted from published data (Heare et al. 2017) (Figure S1). DNA was extracted from gill, and libraries were generated for each sampled oyster using a commercial preparation kit (Illumina, USA) following the manufacturer's recommendations and then sequenced on the NovaSeq PE150 platform (Illumina) as described above.

High‐quality paired‐end reads were mapped to the reference genome using BWA (mem ‐M ‐k 32). SNP calling was performed at the population level using a Bayesian approach implemented in the package SAMtools (Li et al. 2009). The “mpileup” command was used to identify SNPs with the parameters as “‐q 1‐C 50‐t SP‐t DP‐m 2‐F 0.002.” To exclude SNP calling errors caused by incorrect mapping or InDels (Insertions and Deletions), only SNPs with coverage depth ≥ 6, MAF ≥ 0.05 and missing ≤ 0.1 were kept for subsequent population structure analysis. Functional annotation of SNPs was performed using the package ANNOVAR (Version 2013‐05‐20) (Wang et al. 2010). Genotype likelihoods and allele frequencies were calculated for each genomic position of each oyster.

To study the phylogenetic relationship from a genome‐wide perspective for all individuals, a neighbor‐joining (NJ) tree was constructed using the software TreeBest (v1.9.2) (http://treesoft.sourceforge.net/treebest.shtml). Principal component analysis (PCA) was conducted to evaluate population structure using the software GCTA (Yang et al. 2011). The population structure was also investigated using ADMIXTURE (v1.3.0), with the genetic clade numbers being predefined from *K* = 2 to *K* = 7 (Alexander et al. 2009). The most suitable number of ancestral populations was determined by the *K* value with the lowest cross‐validation (CV) error. Linkage disequilibrium (LD) in each population was measured with the correlation coefficient *r* ^2^ calculated using PopLDdecay (v3.30) with MaxDist being set at 100 (Zhang et al. 2019). An average *r* ^2^ value was calculated for pairwise markers in a 500 kb window and then averaged across the genome. Population size (Ne) over historical time was estimated using one representative individual from each population using the pairwise sequentially Markovian coalescent (PSMC) method (version 0.6.4‐r49) (Li and Durbin 2011). A mutation rate per nucleotide per generation (μ) of 0.2 × 10^−8^ and a generation time (g) of 1 year were used to convert the scaled time and population size into real time and size (Li, Dai, et al. 2021).

To identify genomic regions showing selection associated with domestication and/or environmental adaptation, genome‐wide distribution of fixation index (*F*_ST_) values and θπ ratios were calculated for selected group pairs using VCFtools (v0.1.13) (Danecek et al. 2011), with a sliding window size of 100 kb and a step size of 50 kb. Windows with the top 5% *F*_ST_ and log_2_ (θπ ratio) values were considered as candidate outlier regions under selection. All genes located in the outlier windows were extracted and listed, and GO enrichment analysis of these genes was conducted accordingly (Li et al. 2013).

## 3 Results

### 3.1 Genome Sequencing and Assembly

We generated 68.08 Gb (~112×), 815.58 Gb (~1324×) and 69.77 Gb (~114×) sequence data from short‐read shotgun sequencing, long‐read PacBio Sequel II sequencing, and HiC sequencing, respectively, for genome assembly. In addition, a total of 91.98 Gb of transcriptomic sequence data was obtained for genome annotation (Table S3).

**FIGURE 1:**
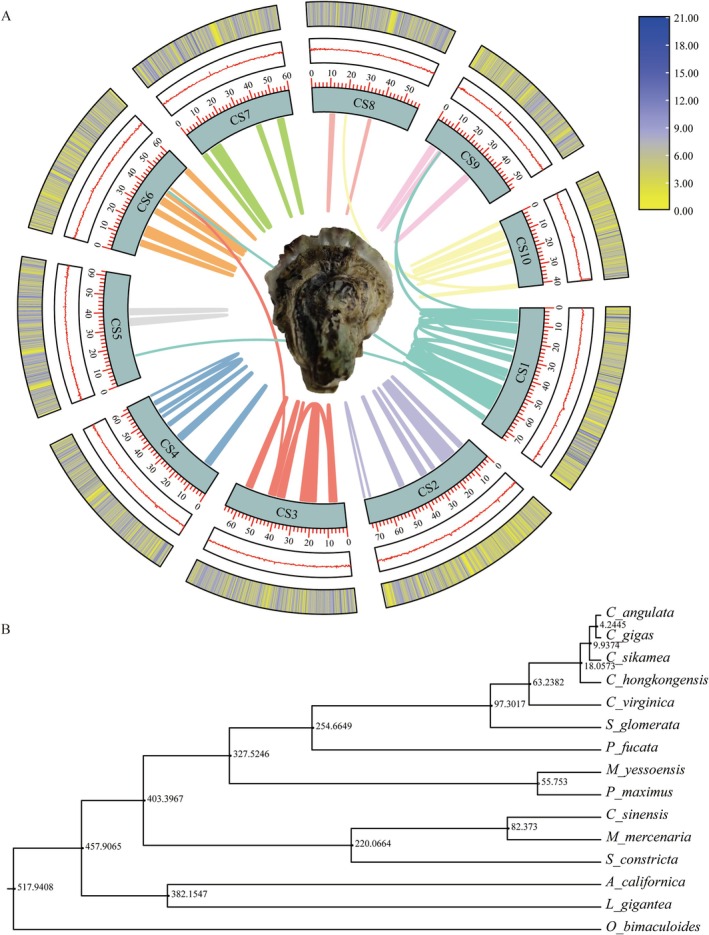
Genome landscape and phylogenetic relationships of *Crassostrea sikamea*. (A) Circular maps of the *C. sikamea* genome with a representative oyster in the center, from inside, (I) synteny among chromosomes within the genome; (II) 10 haploid chromosomes at the Mb scale; (III) GC content ranging from 0 to 1 (red line); and (IV) gene density (yellow to blue lines) on each chromosome. GC content and gene density are presented in sliding windows of 5 kb and 100 kb, respectively. (B) Phylogenetic tree based on 237 strictly single‐copy orthologs from 15 mollusk species. Numbers on at nodes indicate estimated divergence time in million years ago (Mya).

The estimated genome size based on the *k*‐mer (*k*‐mer = 21, depth = 109) distribution was 523.97 Mb, the heterozygosity rate was 3.38%, and the proportion of repetitive sequences was 31.65% based on the *k*‐mer analysis (Figure S2, Table S4). The best contig‐level assembly from Hifiasm software consisted of 282 contigs and 250 scaffolds with a total length of 616.49 Mb and a contig N50 of 4.21 Mb (Figure 1A, Table S5). The chromosome‐level assembly based on HiC sequencing consisted of 27 scaffolds assembled in 10 pseudomolecules corresponding to the 10 chromosomes of *C. sikamea*, and 233 small scaffolds, together composing 99.87% of the bases of the contig‐level assembly (Figure S3). The chromosome‐level assembly had a scaffold N50 of 62.25 Mb (Figure 1A, Tables S5 and S6).

The GC content of the final assembly in a 10 kb window followed a nearly normal distribution without obvious left and right blocking phenomenon for GC depth, indicating high assembly quality (Figure S4). The read mapping rate and coverage were 96.9% and 99.8%, respectively, highlighting the consistency and completeness of the assembly (Table S7). BUSCO analysis indicates that 98.9% of Mollusca conserved genes are completely represented together with 98.6% complete (with 92.6% single and 6.0% duplicated), 0.3% fragmented, and 1.1% missing. The quality parameters of *C. sikamea* genome assembly are high and comparable to several recently published oyster and bivalve genomes (Table 1).

**TABLE 1:** Parameters of *C. sikamea* genome assembly compared to several recently published oyster and bivalve genomes.

	*C. sikamea*	*C. gigas*‐Roslin	*C. gigas*‐Qingdao	*C. hongkongensis*	*C. ariakensis*	*C. virginica*	*S. constricta*	*P. maximus*	*M. coruscus*
Genome size (Mb)	616.5	647.9	586.8	610	613.9	684.7	1332	918.3	1567
GC content (%)	33.4	33.3	—	33.5	33.4	35	—	36.6	32
Contig N50 (Mb)	4.2	1.8	3.1	2.57	7.0	2.0	0.68		1.5
Scaffold N50 (Mb)	62.3	58.5	—	55.63	62.3	75.9	58	44.8	—
BUSCO									
Complete (%)	98.6	95.5	92.5	95.8	92.5	97.2	88.8	94.5	85.5
Single (%)	92.6	93.8	86.4			95.1	85.1	91.2	84.2
Duplicated (%)	6.0	1.7	6.1			2.1	3.7	3.3	1.3
Fragmented (%)	0.3	0.4	0.7	0.8		0.7	4.1	1	0.8
Missing (%)	1.1	3.9	6.8	3.4		2.1	7.2	4.5	13.7
References	Present study	Penaloza et al. (2021)	Qi et al. (2021)	Zhang et al. (2022)	Li et al. (2021)	Puritz et al. (2023)	Dong et al. (2020)	Kenny et al. (2020)	Yang et al. (2021)

### 3.2 Genome Annotation

Overall, tandem repeats and transposable elements (TEs) accounted for 47.02% (289.9 Mb) of the assembled genome. The TEs were dominant and accounted for 45.5% of the genome (Table S8), including 17.62% of DNA transposons and 5.81% of retrotransposons (Table S9). Further, a total of 0.41 Mb ncRNA, including 428 rRNA, 3695 tRNA, 308 miRNA, and 175 snRNA, was predicted and accounted for 0.067% of the assembled genome (Table S10). Gene sets were predicted and merged from ab initio (Augustus, GeneMark and SNAP) and homologous predictions from five other species and RNA‐seq data (PASA, Table S11) using EVM. A total of 37,121 protein‐coding genes were predicted in the *C. sikamea* genome (Table S11), which is similar to that in other published bivalve genomes (Bao et al. 2021; Li, Dai, et al. 2021; Qi et al. 2021; Song et al. 2021; Yang et al. 2021; Puritz et al. 2023; Teng et al. 2023). Gene structure distribution shows similar patterns among five oyster species (Figure S5, Table S12). A total of 36,989 (99.6%) genes could be functionally annotated or had homologues in at least one database (Table S13, Figure S6). We did BUSCO analysis with the mollusca_odb10 database for the predicted protein‐coding genes; in the 5295 BUSCOs, 90.2% were complete (with 82.1% single and 8.1% duplicated), 0.3% were fragmented, and 1.1% missing.

### 3.3 Genome Phylogeny

The protein‐coding genes of the 15 selected species were clustered, and 237 strictly single‐copy orthologs were identified. Maximum likelihood phylogenetic analysis of these single‐copy orthologs indicated that *C. sikamea* diverged from the *Crassostrea angulata* and *C. gigas* clade 9.9 million years ago (Mya) and diverged from *C. hongkongensis* 18.1 Mya (Figure 1B), and the node of three *Crassostrea* oysters in Asia diverged from *C. virginica* in the Atlantic approximately 63.2 Mya.

### 3.4 Genome Synteny

**FIGURE 2:**
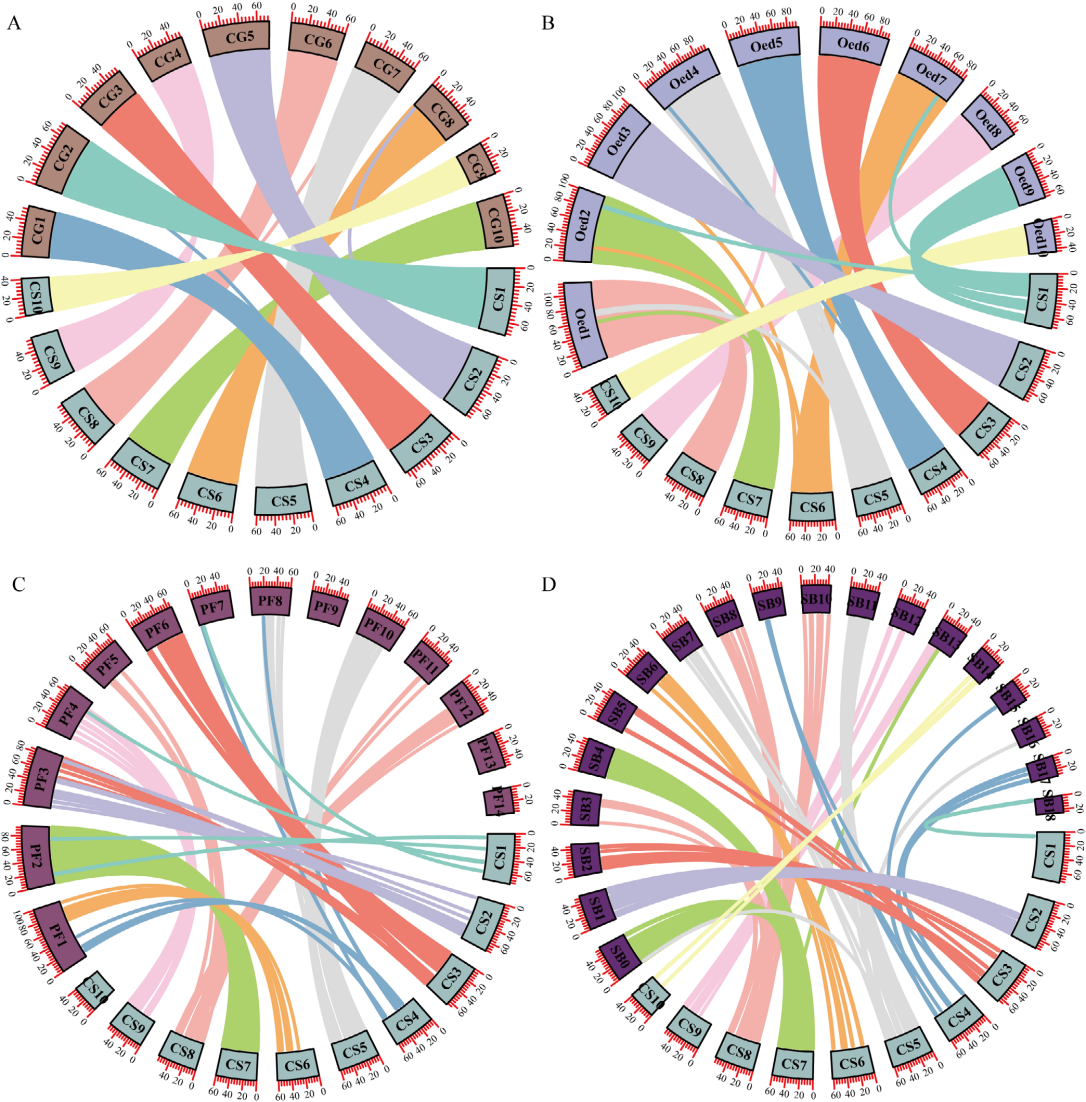
Genome synteny between *C. sikamea* and *C. gigas* (A), *Ostrea edulis* (B), *P. fucata* (*n* = 14) (C), and *Scapharca broughtonii* (*n* = 19) (D). Lines in different colors represent different chromosomes of *C. sikamea*, blocks represent chromosomes, and the scale is at the Mb scale.

Comparisons between *C. sikamea* and three other oyster species with *n* = 10 ( *C. gigas* , *C. virginica* , and *O. edulis* ), two species with *n* = 14 (mussel *M. coruscus* and pearl oyster *P. fucata* ), and two species with *n* = 19 (blood clam *S. broughtonii* and Venus clam *C. sinensis* ) revealed various levels of chromosomal synteny among these bivalves. Synteny among oyster species (*n* = 10) is remarkably high. Between *C. sikamea* and *C. gigas* , 385 collinear blocks were identified, covering 18,286 and 17,315 genes of *C. sikamea* and *C. gigas* , respectively. Of these, the synteny of 382 blocks is completely conserved on the 10 chromosomes, and only 3 blocks were found on different chromosomes (Figure 2A). Between *C. sikamea* and *O. edulis* , 799 collinear blocks were identified covering 13,319 and 12,932 genes of *C. sikamea* and *O. edulis* , respectively. Other than the translocation of a few blocks, all 10 chromosomes were mostly conserved (Figure 2B). Between *C. sikamea* and *C. virginica* , 975 collinear blocks were identified covering 15,861 and 17,957 genes of *C. sikamea* and *C. virginica* , respectively. Five chromosomes are nearly completely conserved between the two species (CS3‐CV4, CS4‐CV1, CS5‐CV3, CS6‐CV8, CS7‐CV2), but chromosomes CV5 corresponded to CS8 and CS9, CS2 corresponded to CV6 and CV7, and CS10 and a part of CS1 corresponded to CV9, and the other part of CS1 is synteny with CV10 (Figure S7A). The discrepancy can be explained by known assembly errors of *C. virginica* assembly (Puritz et al. 2023, see Section 4).

There was less synteny between the *C. sikamea* and pearl oyster *P. fucata* and mussel *M. coruscus* (*n* = 14), blood clam *S. broughtonii*, and Venus clam *C. sinensis* (*n* = 19). Two pairs of chromosomes (CS7‐PF2 and CS9‐PF4) were almost entirely conserved between *C. sikamea* and *P. fucata* (Figure 2C). Whereas four *P. fucata* chromosomes (PF7, PF9, PF13, and PF14) showed no or only one synteny block with the *C. sikamea* chromosomes, and CS10 and CS1 had no or fewer than five synteny blocks with PF chromosomes. Two chromosome fusion events were evident: PF5 and P12 into CS8, and PF8 and PF10 into CS5. Between *C. sikamea* and *M. coruscus*, MC01 and MC08 were fused into CS7, whereas chromosomes MC14, MC12, and MC06 had no more than one collinear block with any one *C. sikamea* chromosome (Figure S7B). Eight of *C. sikamea* chromosomes showed synteny with various chromosomes of *S. broughtonii* with possible fusions of SB0 and SB4 into CS7, SB7, and SB11 into CS5, SB2, and SB5 into CS3, SB3, SB8, and SB10 into CS8, while CS1 and CS10 showed only one or two synteny blocks with *S. broughtonii* chromosomes (Figure 2D). Few synteny blocks were identified between *C. sikamea* and *C. sinensis* , probably due to poor chromosome assembly of the *C. sinensis* genome (Figure S7C). These findings suggest extensive rearrangements, especially fusions and chromosomal loss, have occurred during bivalve evolution leading to oysters, while within the oyster lineage chromosomes are highly conserved.

### 3.5 Population Structure and Demographic History

The whole‐genome resequencing of the 141 Kumamoto oysters from 7 different geographic locations generated 1830 Gb filtered sequence data, with an average mapping rate of 91.56% and an average depth of 16.82 X per individual (Table S14). A total of 66,714,120 raw SNPs were identified in the 141 genomes, and 1,954,312 high‐quality SNPs were retained after stringent quality control. Among them, 1,093,148 (55.9%) were genic, including 366,293 (18.7%) exonic and 726,232 (37.2%) intronic; 598,231 (30.6%) were intergenic, and 221,024 (11.3%) were located in the < 1 kb upstream or downstream ranges of genes (Table S15).

**FIGURE 3:**
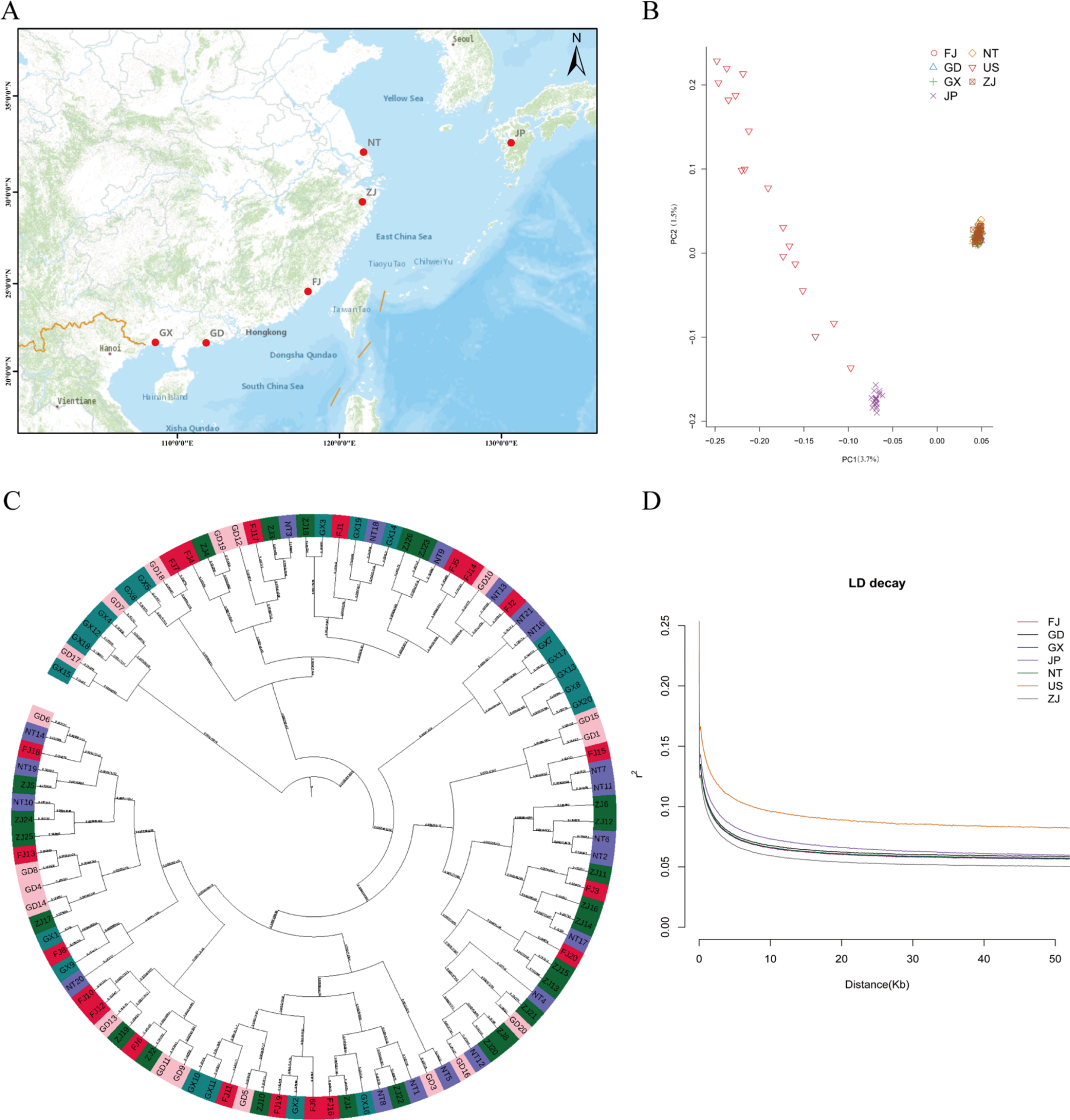
Genomic analysis of *C. sikamea* populations. (A) Sampling sites of natural populations in Asia: FJ, Fujian; GD, Guangdong; GX, Guangxi; JP, Japan; NT, Nantong; ZJ, Zhejiang. (B) Principal component analysis (PCA) of individuals from seven populations. (C) Phylogenetic tree for all resequencing individuals from five sites in China; different colors represent different sites, and the individuals are named with the abbreviation of the sampling site and a serial number. (D) LD decay in 50 kb regions. The horizontal axis is the distance at which LD occurs, and the vertical axis represents the correlation coefficient of LD (*r* ^2^).

The retained SNPs after QC were analyzed using multiple methods to infer the genetic structure of the sampled oysters. In PCA, oysters from the 5 geographic locations in China (FJ, GD, GX, NT, ZJ, Figure 3A) clustered together, while Japanese oysters (JP) formed another distinct cluster (Figure 3B). The cultured oysters from the United States were, on the other hand, loosely clustered and close to the JP population (Figure 3B, Figure S8). The ADMIXTURE analysis also revealed the clear separation between the Chinese and Japanese populations and the close relationship between the Japanese and US populations (*K* = 3, Figure S8A). Additionally, the US population was clustered together with the Japanese population in phylogenetic analysis (Figure S8), consistent with the known introduction of *C. sikamea* from Japan to the United States. Interestingly, oysters from China did not form clusters according to their geographic distribution on the phylogenetic tree (Figure 3C and Figure S10), suggesting a lack of population structure.

The LD decayed rapidly in all three populations, with the estimated LD decreasing from the maximum of 0.25–0.125 in 0.1–0.4 kb for Chinese populations, in 0.8 kb for the Japanese population, and in 2 kb for the US population, and all stabilized at the distance of about 50 kb (Figure 3D and Figure S9). The levels of LD were similar between the Chinese and the Japanese populations, and both were significantly lower than that of the US population, suggesting the existence of a bottleneck in effective population size for the US population.

**FIGURE 4:**
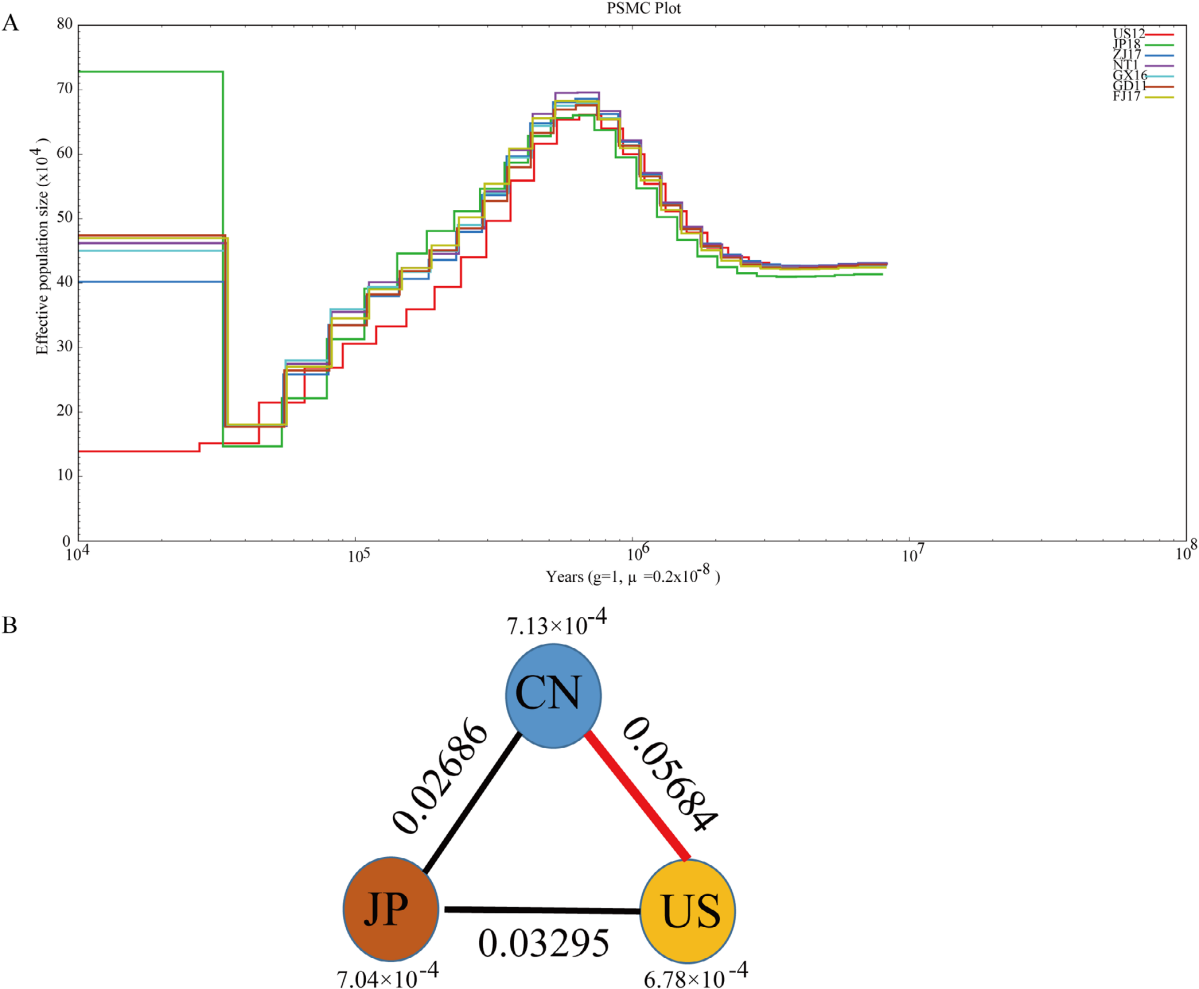
Demographic history and genetic differentiation statistics *F*_ST_ and θπ. (A) Demographic history of the *C. sikamea* inferred by pairwise sequentially Markovian coalescent (PSMC) model. The horizontal axis represents years before present, and the vertical axis represents the effective population size (Ne), with the generation interval as 1 year and mutation rate (μ) = 0.2 × 10^−8^ per nucleotide per generation. (B) Nucleotide diversity (θπ) and genetic divergence (*F*_ST_) across the three geographic populations studied. CN represents all five subpopulations from China. The value of nucleotide diversity is presented next to each site, and pairwise *F*_ST_ is indicated on the lines linking population pairs.

PSMC analysis estimated a peak effective population size of *Ne* = 70 × 10^4^ individuals at ~0.7 Mya for all three *C. sikamea* populations (Figure 4A). The *Ne* value decreased gradually to the lowest level of about 15 × 10^4^ at 50 kya, and then the population expanded at ~20 kya to the effective population size of about 45 × 10^4^ (Figure 4A). The current or most recent *Ne* for the US hatchery population was several folds smaller than that of the natural populations from China and Japan.

No significant differences in nucleotide diversity and fixation index (*F*_ST_) were observed among the five Chinese geographic locations (subpopulations) (Table S16). However, differentiations among all three populations were statistically significant, with the estimated fixation index (*F*_ST_) being 0.05684 between the Chinese and US populations, 0.03295 between the Japanese and US populations, and 0.02684 between the Chinese and Japanese populations (Figure 4B). In addition, the nucleotide diversity (θπ) in the US population (6.78 × 10^−4^) was significantly lower than that in the Chinese populations (7.13 × 10^−4^, *p* = 1.17E‐08) and Japanese population (7.04 × 10^−4^, *p* = 1.94E‐05). No significant differences in nucleotide diversity were detected between the Chinese and Japanese populations (*p* > 0.05) (Figure 4B).

### 3.6 Selection Signatures for Domestication and Environmental Adaptation

**FIGURE 5:**
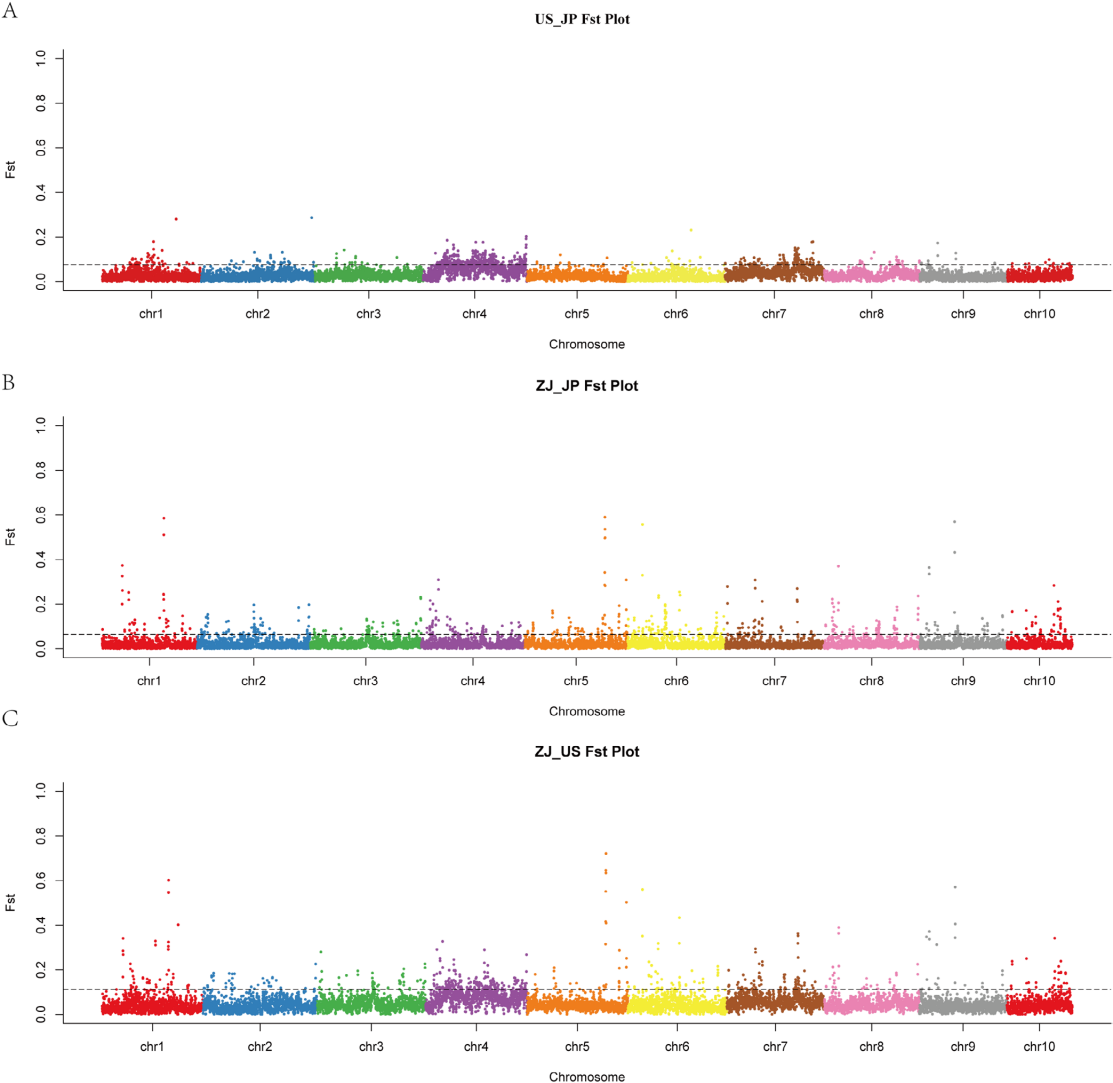
Global genomic *F*_ST_ among three population pairs. The genome‐wide distribution of fixation index (*F*_ST_) values calculated with a slide window of 100 kb and step size of 50 kb for the defined group pairs: US & JP (A), ZJ & JP (B), and US & ZJ (C). The horizontal dashed lines represent the top 5% *F*_ST_ value thresholds as 0.08, 0.06, and 0.11 for (A), (B), and (C), respectively. The ZJ subpopulation was selected to represent the Chinese population to ensure all the compare pairs contain a similar number of individuals.

**FIGURE 6:**
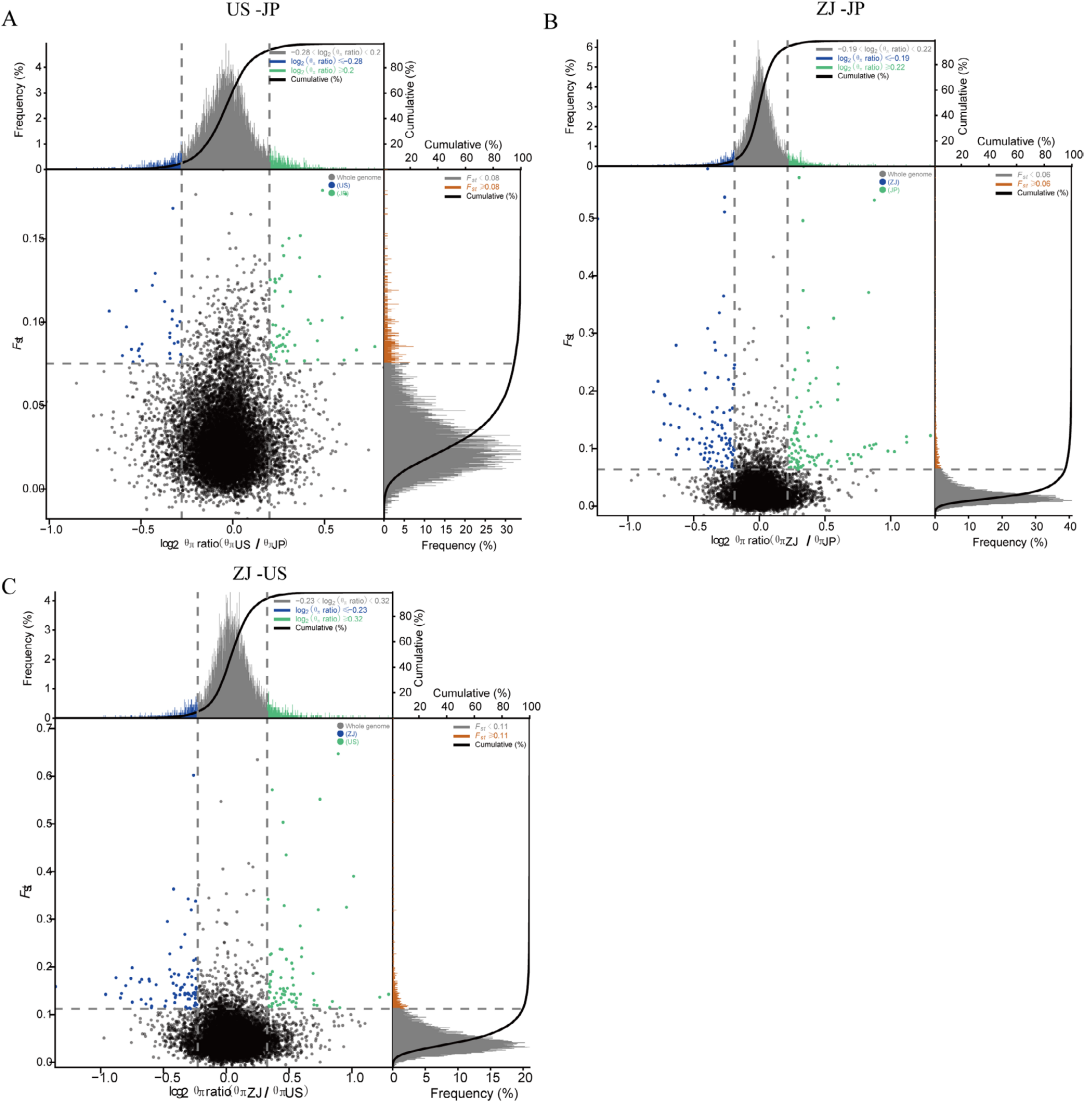
Genome‐wide selective sweeps associated with domestication and environment adaptation. Windows simultaneously fit with intersection of the top 5% of *F*_ST_ values and log_2_ (θπ ratio) as regions with selective sweep signals in a slide window of 100 kb and step size of 50 kb for the defined group pairs: US versus JP (A), ZJ versus JP (B), and US versus ZJ (C). Significant *F*_ST_ thresholds for A, B, and C are ≥ 0.06, 0.06, and 0.11, respectively (top 5%), and log_2_ (θπ ratio) thresholds ≥ 0.2, 0.22, and 0.32 (top 5%, green) or ≤ −0.28, −0.19 and −0.23 (bottom 5%, blue).

**FIGURE 7:**
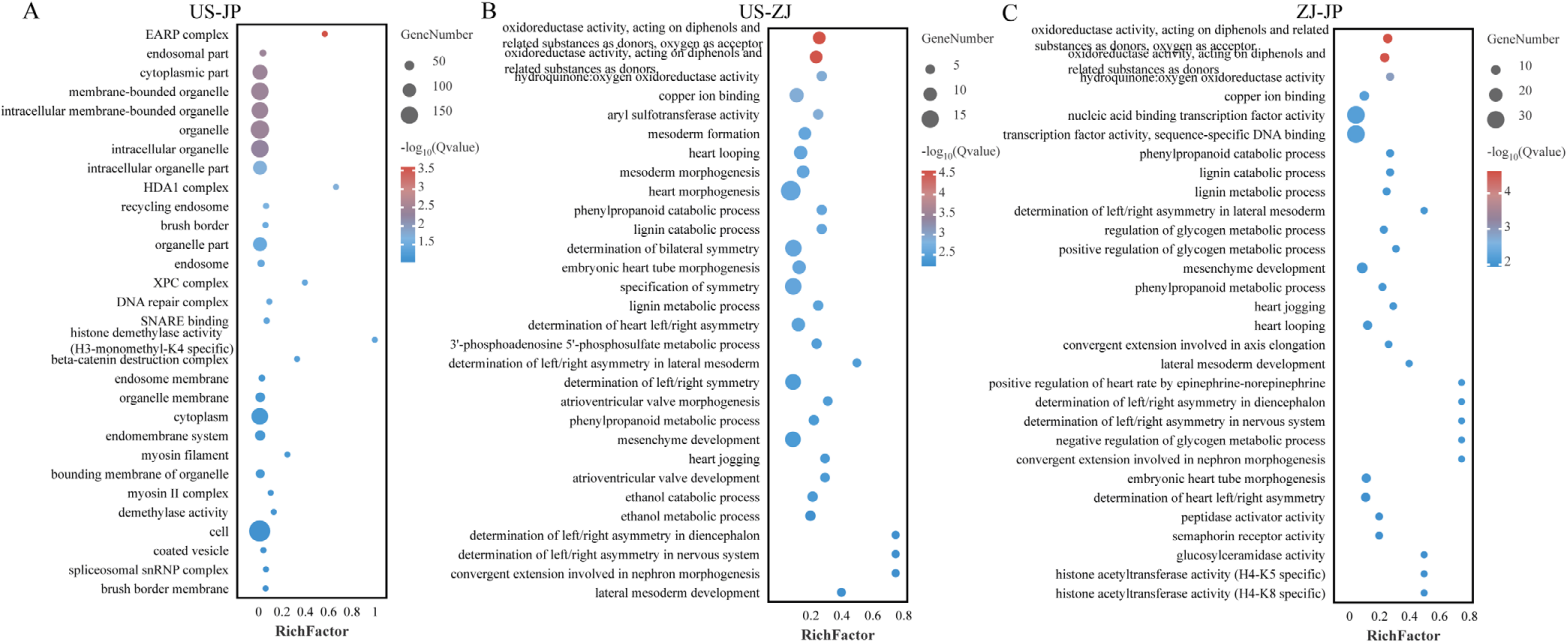
GO enrichment analysis of genes under selective sweep for the defined group pairs: US & JP (A), US & ZJ (B), and ZJ & JP (C). The top 30 enriched GO terms, ranked by Q‐value, are displayed in biological processes, molecular functions, and cellular components and are visualized in different colors.

To identify signatures of domestication or environmental adaptation, pairwise *F*_ST_ and θπ ratios between the US population, JP population, and the ZJ subpopulation from China were calculated. The top 5% windows with the highest *F*_ST_ and θπ values (*F*_ST_ US vs. JP ≥ 0.08, *F*_ST_ US vs. ZJ ≥ 0.11, *F*_ST_ ZJ vs. JP ≥ 0.06) were selected as outlier regions with selective sweep signals for identifying genes under selection (Figures 5 and 6). A total of 402 genes in 76 selected windows were identified as candidate genes under selection in the comparisons between the US and JP populations (Figure S11, Table S17, Table S18), which are listed alphabetically by their NR (Non‐Redundant) gene description (Table S18). GO enrichment analysis revealed that the top enriched GO terms of the identified genes were in the cellular component, including endosome‐associated recycling protein complex, cytoplasmic part, beta‐catenin destruction complex, myosin filament, and molecular function included SNARE binding, histone demethylase activity, demethylase activity (Figure 7A, Table S19). Among them were genes related to growth regulation such as multiple epidermal growth factor‐like, follistatin, and ribosomal proteins, genes involved in stress and immune response such as cytochrome 450, C1q domain containing protein (C1qDC), C‐type lectin, ficolin, and heat shock 70 protein (hsp70)‐binding protein, myeloid differentiation primary response protein (MyD88) and serine protease inhibitors, and genes encoding actin, fibrillin‐1 (FBN1) and myosin heavy chain, and tyrosinase‐like proteins (Table S18). The analysis also identified 768 and 742 candidate genes under selection by comparisons between the Chinese ZJ subpopulation and the JP population and between the ZJ subpopulation and the US population, respectively (Figure S11, Table S17). The top enriched GO terms of these identified genes were oxidoreductase activity, phenylpropanoid catabolic process, lignin metabolic process, copper ion binding, determination of left/right symmetry (Figure 7B,C). Most of the top enriched GO terms between the ZJ vs. JP and ZJ vs. US were shared, and the most enriched genes included those encoding NADH dehydrogenase, glutathione S‐transferase (GST), multicopper oxidase, glucose‐methanol‐choline (GMC) oxidoreductase, l‐ascorbate oxidase, laccase, cytochrome P450, fucolectin‐like genes, collagen alpha, HSP70s, C1qDC, heavy metal‐binding protein, T‐box transcription factor, and zinc‐finger proteins (Table S18).

## 4 Discussion

The Kumamoto oyster has a wide distribution in Asia Pacific and is an important aquaculture species. Its range has recently expanded northward along the coast of China because of global warming (Hu and Dong 2022). We know little about the biology and adaptive potential of this species partly because of the lack of genomic information and resources. Here, we present a chromosome‐level assembly of the *C. sikamea* genome and genomic analyses of wild and captive populations that provide insights into the genome biology of this species and bivalve evolution.

### 4.1 Genome Assembly

The quality of Kumamoto oyster genome assembly is high and comparable to several recently published oyster and bivalve genomes. The assembled genome size (616.5 Mb) was comparable to that of other oyster species (Table 1, 587–685 Mb), with the assembly larger than the estimated genome size based on the *k*‐mer distribution analysis (524 Mb). The contig and scaffold N50 sizes are larger than most published oyster genomes (Li, Dai, et al. 2021; Penaloza et al. 2021; Qi et al. 2021; Puritz et al. 2023). The 98.6% complete BUSCOs on the genome, 96.88% read mapping rate, and 99.8% genome coverage rate of short reads against the genome indicated a high level of completeness (Tables 1 and Table S7). The genome contains some redundancy as indicated by the complete but duplicated genes (6.0%) and the larger than expected genome size. Heterozygosity rate (3.38%, estimated from *k*‐mer distribution) in Kumamoto oyster genome is the highest among bivalves, especially oysters, where the Portuguese oyster *C. angulata* and estuary oyster *C. ariakensis* have heterozygosity rates of 2.6% and 1.3%, respectively (Wu et al. 2021; Teng et al. 2023). Compared with other *Crassostrea* oysters, *C. sikamea* is known to occupy higher tidal positions and faces harsher environmental conditions. Its high heterozygosity or genetic diversity could be a genomic adaptation for *C. sikamea* to thrive in the unpredictable environments of high intertidal zones (Guo et al. 2018). The number of protein coding genes was predicted to be 37,121, which is comparable to that of other bivalves (Song et al. 2021; Puritz et al. 2023).

### 4.2 Karyotypic Evolution

Synteny analysis revealed remarkable chromosome conservation within Ostreidae that diverged for ~180 million years (Li, Kou, et al. 2021). The quality of genome assembly is vital to various downstream applications, especially karyotype evolution analysis (Sun et al. 2021). A recent work assessed the quality of the latest Pacific oyster genome assembly and found that apparent misassemblies accounted for only 0.08% of the genome (Qi et al. 2021). Except for 3 small blocks (out of 385), all 10 chromosomes of *C. sikamea* exhibited one‐to‐one match with *C. gigas* chromosomes, without any major chromosomal rearrangements (Figure 2A). This result suggests that chromosomes of the two species are almost completely conserved and both assemblies are of high quality. The differences between *C. sikamea* and *C. virginica* are consistent with known errors of the *C. virginica* assembly; for example, chromosomes CV5 and CV9 contained parts of other chromosomes (Puritz et al. 2023). Further, no major chromosome rearrangement was observed between *C. sikamea* and *O. edulis* that have diverged for ~180 million years (Li, Kou, et al. 2021). Similarly, high levels of synteny have been observed between *C. gigas* and *O. edulis* (Gundappa et al. 2022). Oysters are known to have a conserved haploid number (*n* = 10) and karyotype (Wang et al. 2004; Guo et al. 2018). Results of this study suggest that not only is karyotype conserved; gene content and synteny of the chromosomes are also conserved.

Karyotypes of some bivalve molluscs are known to be highly conserved, with the yesso scallop karyotype closely resembling the ancestral bilaterian karyotype (Wang et al. 2017). Both fossil records and phylogenetic analyses have indicated that clams and scallops with *n* = 19 chromosomes emerged first, followed by the emergence of mussels and pearl oysters with *n* = 14, and then oysters with *n* = 10 (Guo et al. 2018). Our analyses confirm that, while the oyster karyotype is highly conserved, significant chromosomal rearrangements have occurred during bivalve evolution, leading to oysters, highlighting the variable rates of chromosomal evolution (Bickham 1981). Several oyster chromosomes (CS5, CS8, CS7) clearly evolved through fusion of two or more chromosomes. Several chromosomes of *P. fucata* and *S. broughtonii* show little or no synteny with *C. sikamea* chromosomes, suggesting they might be lost during evolution. These results provide evidence of extensive chromosomal fusion and loss during bivalve evolution leading to oysters, while demonstrating the near‐complete conservation of chromosomes within the oyster lineage. Further studies are needed to understand the evolutionary significance of these chromosomal rearrangements during bivalve evolution and the apparent lack of chromosomal rearrangements within the oyster lineage. Several chromosomes showed no significant synteny between *C. sikamea* and *P. fucata*, which begs the question of whether these chromosomes are more important in determining the unique characteristics of each species or creating barriers to gene flow.

The near‐complete conservation of 10 oyster chromosomes over 180 million years is remarkable, considering the active chromosomal rearrangements during Metazoan evolution. For example, Carnivora mammals that diverged for ~80 million years exhibited diverse haploid chromosome numbers ranging from 15 to 37 (O'Brien et al. 1999). It has been suggested that increased chromosome number creates more variation, which is advantageous for stressful or heterogeneous environments (Qumsiyeh 1994; Qumsiyeh and Handal 2022). Compared with free‐living clams and scallops that live subtidally, oysters face more environmental challenges. The fact that oysters have fewer and more highly conserved chromosomes is surprising and argues for alternative explanations. We hypothesize that the ancestral karyotype of bivalves has 19 chromosomes, like that of most clams and scallops, and extensive chromosomal rearrangements and loss occurred during bivalve evolution leading to oysters, resulting in a final karyotype of 10 chromosomes. The remarkable ability of oysters to cope with stressful environments may have developed early during the emergence of oysters because of the significant chromosomal rearrangements. After early canalization (Bickham 1981) leading to the emergence of oysters with 10 chromosomes, large population size kept the karyotype stable, and other evolutionary forces became dominant in the divergence of oysters.

Synteny analysis examines synteny in small homologous gene blocks. It is possible that, while gene content and small synteny blocks are conserved on all 10 chromosomes, significant intrachromosomal rearrangements (translocations and inversions) exist and are not detected due to the methods used. Further, it is possible that since the emergence of the oyster lineage and early canalization of the karyotype, subsequent divergence and adaptation involved intrachromosomal rearrangement or tandem duplications of genes or chromosomal fragments (Guo et al. 2015, 2018). Additional studies are needed to delineate gene synteny at fine scales and study the phenotypic effects of chromosomal rearrangements in populations or closely related species such as *C. gigas* and *C. angulata* (Teng et al. 2023). Nevertheless, the remarkable conservation of chromosomes over 180 million years of gene content and order on oyster chromosomes suggests that such conservation is not accidental but has evolutionary significance. It is not inconceivable that certain sets of genes function more efficiently by being clustered on the same chromosome and in a certain order, and chromosomal rearrangements would affect phenotype and fitness (Damas et al. 2021). It has been shown that certain gene families are preferentially distributed on the same chromosome. In *C. virginica* , for example, 22 of the 23 expanded polyalanine containing protein (Pacp) genes that are involved in shell formation are found on chromosome 4 (Zeng and Guo 2022). In the hard clam, 59 of the 159 inhibitors of apoptosis genes are located on one of the 19 chromosomes (Song et al. 2021). A significant role of chromosomal organization in evolution, if confirmed, would be another extension to the allele‐focused Modern Synthesis.

### 4.3 Differentiation Related to Domestication and Environmental Adaptation

The population structure based on the whole‐genome SNPs shows that the five Chinese subpopulations are clustered together, away from the other two populations from Japan and the United States. PSMC analysis indicated a geographical isolation time about 20 kya between China and Japan populations. Sekino et al. (2012) postulated that the ancestral *C. sikamea* population(s), which most likely inhabited the paleo‐ECS/YS estuarine zones during glacial cycles, colonized the Ariake Sea in Japan fairly recently (at least after 15 kyr BP), based on the presence of a few high‐frequency COI haplotypes and a certain number of rare haplotypes in the Japan population. Kumamoto oysters are considered an estuary species, with patchy distribution in the Ariake Sea in Japan (Hedgecock et al. 1999; Camara et al. 2009). Our results indicate that the global *F*_ST_ values were relatively low between the Chinese and Japanese populations (*F*_ST_ = 0.027, *p* < 0.001; Figure 4). Whereas analysis based on the mitochondrial COI gene shows that the Japanese and American populations have significantly lower nucleotide and haplotype diversities than the Chinese population, together showing much higher *F*_ST_ with the Chinese populations (*F*_ST_ > 0.2, Xuan et al. 2024). Elevated differentiation in three mitochondrial genes was also found among China, Japan, and Korea populations, which was attributed to possibly localized larval retention, isolation, and genetic drift (Sekino et al. 2012). However, Chinese populations showed no significant genetic differentiation and no IBD pattern based on mitochondrial genes (Hu et al. 2018), reduced‐representation genome sequencing (Hu and Dong 2022), or whole‐genome resequencing (the present study, Figure 3C, Figure S10, Table S16). Analysis of mitochondrial DNA or whole‐genome sequences in *C. gigas* both indicate no significant genetic differences between populations in China and Japan, suggesting extensive gene flow (Sekino et al. 2012; Hu et al. 2022). In contrast, the estuary oyster *C. ariakensis* showed clearly patchy distribution and significant divergence in both mitochondrial and genome‐wide variation (Li, Dai, et al. 2021). Compared to *C. gigas* , *C. ariakensis* tends to inhabit subtidal zones and estuaries of lower salinity, resulting in a relatively restricted distribution range for its planktonic larvae and thereby substantially reduced gene flow among its populations. It is apparent that *C. sikamea*, due to the adaptation to moderate salinity, has a widespread distribution along the southern coast of China and maintains strong gene flow (Hu et al. 2018). Results of this study suggest that the divergence of *C. sikamea* in China and Japan, as measured by genome‐wide SNPs, is significant but small, possibly due to recent isolation.

The introduced population of Kumamoto oyster in the United States provided an interesting case for studying domestication or adaptation to a new environment with much less variation in temperature. The introduction lasted for about 10 years (1947–1959), and only a small proportion of the transported seedlings were Kumamoto oysters (Sekino 2009). Nucleotide diversity analysis showed that the American population was significantly lower than the Chinese and Japanese populations (Figure 4). Higher levels of LD also indicated that the American population has undergone artificial selection or domestication processes compared with natural populations, similar to what was found in artificially selected scallop populations (Wang et al. 2021, 2023). Population structure analysis illustrated the scattered distribution of the American population, which indicates divergence since its introduction to the United States about 80 years ago (Sekino 2009). Such divergence may be the result of isolated reproduction at different hatcheries or multiple introduction events. Several hatchery lines and at least one recent introduction have been reported (Hedgecock et al. 1993; Camara et al. 2009). Nevertheless, our results clearly indicate that the American population is closer to the Japanese population than to Chinese population, confirming its Japanese origin. One study has reported a small effective population size in one of the hatchery stocks, which may lead to inbreeding, genetic drift, and founder effects (Hedgecock et al. 1993). Founder effects could explain the frequency changes of a non‐synonymous mutation (C/A, chr4‐30,069,939) in the encoding region of FBN1. The A frequency in the Chinese and Japanese populations is 0% and 2.8%, respectively, while the frequency increased greatly to 41% in the American population. However, it is also possible that the A allele was subjected to positive selection during domestication or adaptation to new environments in the United States (see below). A similar phenomenon was observed between wild and hatchery populations in other oysters or scallops (Sutherland et al. 2020, 2023; Puritz et al. 2023).

Domestication and/or environmental adaptation led to significant differentiation between the American and Japanese populations, and the *F*_ST_ between the American and Japanese populations is greater than that between the Chinese and Japanese populations. This finding is consistent with a recent study in the eastern oyster, which showed that rapid genomic changes can occur during domestication or closed reproduction (Zhao et al. 2024). Hatchery‐propagated populations had higher interindividual relatedness suggesting family structure, which was also observed in *C. virginica* (Puritz et al. 2022).

Genes with possible signatures of a selective sweep in the American population included two FBN‐1 and two actins. FBN‐1 is a glycoprotein and has been shown to be closely related to height in humans (Asgari et al. 2020) and may help in the rapid body swelling of leeches after bloodsucking (Zheng et al. 2023), and three of the four fibrillin genes are associated with growth regulation in the Pacific oysters (Jiao et al. 2021). FBN1 also interacts with numerous microfibril‐associated proteins, growth factors, and cell membrane receptors, thereby mediating a wide range of biological processes such as cell survival, proliferation, migration, and differentiation and regulating the fate of skeletal stem cells (Li et al. 2024; Smaldone et al. 2016). Actins are part of the cytoskeleton and are related to cell growth and mobility, including muscle contraction. Three genes coding for myosin heavy chains were found in regions with selection signals in *C. sikamea* between the hatchery and natural populations (Table S18). Interestingly, three myosin heavy chain genes and many genes related to muscle function were also identified as associated with domesticated populations of *C. virginica* (Zhao et al. 2024). Myosin heavy chains, together with actin filaments, are involved in muscle contraction (Li et al. 2019; Li, Kou, et al. 2021). It is possible that hatchery production and aquaculture create new lifestyles that require different levels or forms of muscle function and therefore cause the selection of genes related to muscle function. For example, larvae in aerated culture tanks at high density may have to swim more, and single oysters in culture cages without secure attachment to substrate may need stronger adductor muscles to manage their valves. One of the myosin heavy chain genes implicated in this study has a non‐muscle form and may function in cell movement, such as phagocytosis. Its homolog in *C. virginica* is differentiated in domesticated populations (Zhao et al. 2024), highly expressed in hemocytes, and associated with *Perkinsus marinus* resistance (Wang et al. 2025). Further studies are needed to confirm if these genes are important for the domestication and adaptation of oysters to hatchery/culture conditions. Other genes under possible selection included cytochrome 450, C1qDC, C‐type lectin, hsp70‐binding protein, MyD88, and serine protease inhibitors that might be related to immune and stress adaptation. The Kumamoto oyster is naturally distributed in subtropical areas, while the American population is produced in the hatchery and cultured in high‐latitude regions (47.22° N), where the summer temperature is significantly lower than in its native habitat, and the winter temperature (6.9°C in February) is similar to that of Japan (10.6°C in February). We postulate that the lack of summer stress in America may explain why some environmental adaptation genes were enriched in the US versus Japan comparison. However, stress from the hatchery environment, such as high culture densities and a higher risk of early life disease, may add selection pressure to related genes.

In contrast, many genes related to stress response were identified in regions showing selection signals between the Chinese and Japanese (plus its derived US) populations. They included genes related to oxidation–reduction, detoxification, and protein chaperones such as NADH dehydrogenase, GSTs, multicopper oxidase, GMC oxidoreductase, l‐ascorbate oxidase, laccase, cytochrome P450, and HSP70s. These results indicate that genes related to stress response are important for the local adaptation of the Chinese and Japanese populations. Environmental stress is a key driver for oyster adaptation. Many gene families related to stress and immune response are expanded in oysters for adaptation to highly variable environments in estuaries and intertidal zones (Zhang et al. 2012, 2015; Guo et al. 2015).

The ridged shell is a unique characteristic of *C. sikamea*, and the American population has more pronounced ridges on its shells (figure 2 in Sekino 2009) than the Chinese population. It has been shown that ridges on the left shell are stably inherited but recessive. Interestingly, T‐box transcription factors, including Tbx4 and Tbx5 were identified in regions under selection, which control the specific shaping of hindlimbs and forelimbs in vertebrates (Khan et al. 2002; Don et al. 2016; Lin et al. 2016). Thus, we speculate that these genes might function in ridge formation; however, the exact function of these Tbx genes needs to be verified in future study.

In summary, this study generated a high‐quality chromosome‐level genome assembly for Kumamoto oyster *C. sikamea*. Synteny analysis revealed significant chromosomal rearrangements during bivalve evolution leading to oysters, but remarkable conservation of all 10 oyster chromosomes over ~180 million years, making oysters a good model to study chromosomal evolution, whose mechanism and evolutionary significance are not well understood. Population analysis confirmed the Japanese origin of the US population, and no population structure was found in Chinese populations, suggesting strong gene flow. Selection sweep analysis identified genes under possible selection, including genes related to muscle function that may be important for oyster domestication and genes related to stress response that are critical for local environmental adaptation. This study also provides useful genomic resources for comparative genomics and the genetic improvement of cultured Kumamoto oysters.

## Conflicts of Interest

The authors declare no conflicts of interest.

## Supporting information


**Figure S1** Seawater temperature in seven populations/subpopulations. Asia locations were shown with satellite data (2010–2019) and US location with data during Sep, 2013 to Aug, 2014 in Oyster Bay which is near to the US population cultured region, the Dabob bay, from reference (Heare 2017).
**Figure S2** Genome size estimation based on the *k*‐mer, according to the genome survey results, Depth = 109 is the main peak, and the genome size was calculated by the formula (Genome size = Kmer‐number/depth) is about 523,972,884 bp. Depth = 54 (approximately 1/2 of the main peak position) and Depth196 are considered to be caused by genome duplication.
**Figure S3** Heatmap of genome‐wide all‐by‐all Hi‐C interaction of all the chromosomes, the horizontal and vertical coordinates represent chromosomes, and the darker the color in the diagram, the stronger the interaction signal.
**Figure S4** Genome assessment based on GC content, GC Depth, mapping rate and coverage. The figure shows the distribution of GC in genome assembly sequences. The horizontal axis represents the GC content, which is calculated as a 10 kb window. The vertical axis represents the proportion of the number of windows corresponding to the GC content to the total number of windows (A). The figure shows the GC Depth scatter plot, with GC content on the horizontal axis and Depth on the vertical axis. These two values are calculated in sequence using a 10 Kb window. There is no obvious left and right block phenomenon in the GC Depth diagram, indicating no contamination and high assembly quality (B). The mapping rate, coverage, and depth distribution after genome assembly sequence alignment, with the horizontal axis representing the sequencing depth value, which is calculated in sequence using a 10 Kb window(C).
**Figure S5** Comparison of gene structure including gene length distribution (1), exon length distribution (2), CDS length distribution (3), intron length distribution (4), and exon number distribution (5) among related species (Cs referred to Crassostrea sikamea).
**Figure S6** Venn map for comparison of annotated genes in NT, NR, Uniprot‐BLASTX, and UniprotBLASTP databases in NCBI.
**Figure S7** Chromosome synteny between *C. sikamea* and oyster *C. virginica* (A), mussel *M. coruscus* (*n* = 14) (B), and clam *C. sinensis* (*n* = 19) (C).
**Figure S8** Genetic admixture structure (A) and phylogenetic tree (B) for 141 resequencing individuals in seven populations. These results was inferred using the 1,954,312 high quality filtered SNPs, yellow and green color represent individuals from Japan and US, respectively in (B).
**Figure S9** Linkage disequilibrium (LD) decay for seven populations/subpopulations. The horizontal axis represents the distance at which LD occurs, while the vertical axis represents the correlation coefficient of LD (*r* ^2^), vertical dashed line represent corresponding distance for LD decay to half of its maximum valve.
**Figure S10** Population structure analysis for five Chinese populations, PCA (A), and population structure (B).
**Figure S11** Venn map for shared genes between ZJ with JP, US with JP and US with ZJ.

**Table S1** Fifteen molluscan species used for phylogenetic analysis and sources for genome data.
**Table S2** Location information for *C. sikamea* populations sampled from China, Japan and the US.
**Table S3** Summary statistics of sequencing data used for genome assembly annotation.
**Table S4** *K*‐mer statistics from *C. sikamea* genome survey analysis.
**Table S5** Statistics for contig level assembly of *C. sikamea* genome.
**Table S6** Statistics for chromosome level assembly of *C. sikamea* genome.
**Table S7** Mapping and coverage rate based on whole‐genome short sequence reads.
**Table S8** Repeat content in *C. sikamea* genome predicted with different methods.
**Table S9** Types and proportion of different transposable elements in *C. sikamea* genome.
**Table S10** Types and proportion of noncoding RNA in *C. sikamea* genome.
**Table S11** Statistics for gene prediction by different methods.
**Table S12** Statistics for *C. sikamea* genes predicted with different organisms based on homologous genes in related species.
**Table S13** Gene homologues annotated in different databases.
**Table S14** Mapping rate and average depth of resequencing data from different populations.
**Table S15** Genome‐wide SNP annotation and statistics after filtering.
**Table S16** Population fixation index (*F*_ST_) data for seven populations based on genome‐wide SNPs.
**Table S17** Blocks showing selection sweep signals and associated genes summary from different comparisons.

**Table S18** Selected genes list between different population pairs.

**Table S19** GO enrichment for genes list between different population pairs.

## Data Availability

All raw genome and transcriptome sequencing data used for genome assembly and annotation, as well as resequencing data, have been deposited in the NCBI (accession number PRJNA1064429) and will be available after the manuscript is accepted for publication.

## References

[eva70100-bib-0001] Alexander, D. H. , J. Novembre , and K. Lange . 2009. “Fast Model‐Based Estimation of Ancestry in Unrelated Individuals.” Genome Research 19: 1655–1664. 19648217 10.1101/gr.094052.109 PMC2752134

[eva70100-bib-0002] Asgari, S. , Y. Luo , A. Akbari , et al. 2020. “A Positively Selected FBN1 Missense Variant Reduces Height in Peruvian Individuals.” Nature 582: 234–239. 32499652 10.1038/s41586-020-2302-0 PMC7410362

[eva70100-bib-0003] Ashburner, M. , C. A. Ball , J. A. Blake , et al. 2000. “Gene Ontology: Tool for the Unification of Biology. The Gene Ontology Consortium.” Nature Genetics 25: 25–29. 10.1038/75556. 10802651 PMC3037419

[eva70100-bib-0004] Banks, M. , D. McGoldrick , W. Borgeson , and D. Hedgecock . 1994. “Gametic Incompatibility and Genetic Divergence of Pacific and Kumamoto Oysters, *Crassostrea gigas* and *C. sikamea* .” Marine Biology 121: 127–135.

[eva70100-bib-0201] Bao, Y. , Q. Zeng , J. Wang , et al. 2021. “Genomic Insights Into the Origin and Evolution of Molluscan Red‐Bloodedness in the Blood Clam *Tegillarca granosa* .” Molecular Biology and Evolution 38, no. 6: 2351–2365. 33528571 10.1093/molbev/msab030 PMC8136487

[eva70100-bib-0005] Bayne, B. L. 2017. “Chapter 1—Phylogeny.” In Developments in Aquaculture and Fisheries Science, edited by B. Bayne , 1–46. Elsevier.

[eva70100-bib-0006] Beck, M. , R. Brumbaugh , L. Airoldi , et al. 2011. “Oyster Reefs at Risk and Recommendations for Conservation, Restoration, and Management.” Bioscience 61: 107–116.

[eva70100-bib-0007] Bickham, J. W. 1981. “Two‐Hundred‐Million‐Year‐Old Chromosomes: Deceleration of the Rate of Karyotypic Evolution in Turtles.” Science 212: 1291–1293. 17738838 10.1126/science.212.4500.1291

[eva70100-bib-0008] Birney, E. , M. Clamp , and R. Durbin . 2004. “GeneWise and Genomewise.” Genome Research 14: 988–995. 15123596 10.1101/gr.1865504 PMC479130

[eva70100-bib-0009] Boutet, I. , H. J. A. Monteiro , L. Baudry , et al. 2022. “Chromosomal Assembly of the Flat Oyster (*Ostrea edulis* L.) Genome as a New Genetic Resource for Aquaculture.” Evolutionary Applications 15: 1730–1748. 36426129 10.1111/eva.13462 PMC9679248

[eva70100-bib-0010] Camara, M. , J. Davis , M. Sekino , et al. 2009. “The Kumamoto Oyster *Crassostrea sikamea* Is Neither Rare nor Threatened by Hybridization in the Northern Ariake Sea, Japan.” Journal of Shellfish Research 27: 313–322.

[eva70100-bib-0011] Cao, M. , K. Mao , Y. Yan , et al. 2021. “A New Global Gridded Sea Surface Temperature Data Product Based on Multisource Data.” Earth System Science Data 13: 2111–2134.

[eva70100-bib-0012] Chen, C. , H. Chen , Y. Zhang , et al. 2020. “TBtools: An Integrative Toolkit Developed for Interactive Analyses of Big Biological Data.” Molecular Plant 13: 1194–1202. 32585190 10.1016/j.molp.2020.06.009

[eva70100-bib-0013] Cheng, H. Y. , G. T. Concepcion , X. W. Feng , H. W. Zhang , and H. Li . 2021. “Haplotype‐Resolved de Novo Assembly Using Phased Assembly Graphs With Hifiasm.” Nature Methods 18: 170. 33526886 10.1038/s41592-020-01056-5 PMC7961889

[eva70100-bib-0014] Damas, J. , M. Corbo , and H. A. Lewin . 2021. “Vertebrate Chromosome Evolution.” Annual Review of Animal Biosciences 9: 1–27. 33186504 10.1146/annurev-animal-020518-114924

[eva70100-bib-0015] Danecek, P. , A. Auton , G. Abecasis , et al. 2011. “The Variant Call Format and VCFtools.” Bioinformatics 27: 2156–2158. 10.1093/bioinformatics/btr330. 21653522 PMC3137218

[eva70100-bib-0016] Don, E. K. , T. A. de Jong‐Curtain , K. Doggett , et al. 2016. “Genetic Basis of Hindlimb Loss in a Naturally Occurring Vertebrate Model.” Biology Open 5: 359–366. 26892237 10.1242/bio.016295 PMC4810746

[eva70100-bib-0202] Dong, Y. H. , Q. F. Zeng , J. F. Ren , et al. 2020. “The Chromosome‐Level Genome Assembly and Comprehensive Transcriptomes of the Razor Clam (*Sinonovacula constricta*).” Frontiers in Genetics 11: 664. 32733535 10.3389/fgene.2020.00664 PMC7358530

[eva70100-bib-0017] Edgar, R. C. 2004. “MUSCLE: Multiple Sequence Alignment With High Accuracy and High Throughput.” Nucleic Acids Research 32: 1792–1797. 15034147 10.1093/nar/gkh340 PMC390337

[eva70100-bib-0018] Emms, D. M. , and S. Kelly . 2019. “OrthoFinder: Phylogenetic Orthology Inference for Comparative Genomics.” Genome Biology 20: 238. 31727128 10.1186/s13059-019-1832-y PMC6857279

[eva70100-bib-0019] FAO . 2024. The State of World Fisheries and Aquaculture 2024. FAO. 10.4060/cc3141en.

[eva70100-bib-0020] Friedman, C. S. , K. S. Reece , B. J. T. Wippel , et al. 2020. “Unraveling Concordant and Varying Responses of Oyster Species to *Ostreid Herpesvirus* 1 Variants.” Science of the Total Environment 739: 139752. 32846506 10.1016/j.scitotenv.2020.139752

[eva70100-bib-0021] Grabowski, J. H. , R. D. Brumbaugh , R. F. Conrad , et al. 2012. “Economic Valuation of Ecosystem Services Provided by Oyster Reefs.” Bioscience 62: 900–909.

[eva70100-bib-0203] Griffiths‐Jones, S. , S. Moxon , M. Marshall , A. Khanna , S. R. Eddy , and A. Bateman . 2005. “Rfam: Annotating Non‐Coding RNAs in Complete Genomes.” Nucleic Acids Research 33: D121–D124. 15608160 10.1093/nar/gki081 PMC540035

[eva70100-bib-0022] Guindon, S. , J.‐F. Dufayard , V. Lefort , M. Anisimova , W. Hordijk , and O. Gascuel . 2010. “New Algorithms and Methods to Estimate Maximum‐Likelihood Phylogenies: Assessing the Performance of PhyML 3.0.” Systematic Biology 59: 307–321. 20525638 10.1093/sysbio/syq010

[eva70100-bib-0023] Gundappa, M. K. , C. Penaloza , T. Regan , et al. 2022. “Chromosome‐Level Reference Genome for European Flat Oyster ( *Ostrea edulis* L.).” Evolutionary Applications 15: 1713–1729. 36426132 10.1111/eva.13460 PMC9679249

[eva70100-bib-0024] Guo, X. 2009. “Use and Exchange of Genetic Resources in Molluscan Aquaculture.” Reviews in Aquaculture 1: 251–259.

[eva70100-bib-0025] Guo, X. 2021. “Genetics in Shellfish Culture.” In Molluscan Shellfish Aquaculture: A Practical Guide, edited by S. E. Shumway , 393–413. 5M Books Ltd.

[eva70100-bib-0204] Guo, X. , J. B. Puritz , Z. Wang , et al. 2023. “Development and Evaluation of High‐Density SNP Arrays for the Eastern Oyster *Crassostrea virginica* .” Marine Biotechnology 25: 174–191. 36622459 10.1007/s10126-022-10191-3

[eva70100-bib-0026] Guo, X. , S. Ford , and F. Zhang . 1999. “Molluscan Aquaculture in China.” Journal of Shellfish Research 18: 19–31.

[eva70100-bib-0027] Guo, X. , Y. He , L. Zhang , C. Lelong , and A. Jouaux . 2015. “Immune and Stress Responses in Oysters With Insights on Adaptation.” Fish & Shellfish Immunology 46: 107–119. 25989624 10.1016/j.fsi.2015.05.018

[eva70100-bib-0028] Guo, X. , C. li , H. Wang , and Z. Xu . 2018. “Diversity and Evolution of Living Oysters.” Journal of Shellfish Research 37: 755–771. 10.2983/035.037.0407.

[eva70100-bib-0029] Haas, B. J. , S. L. Salzberg , W. Zhu , et al. 2008. “Automated Eukaryotic Gene Structure Annotation Using EVidenceModeler and the Program to Assemble Spliced Alignments.” Genome Biology 9: R7. 18190707 10.1186/gb-2008-9-1-r7 PMC2395244

[eva70100-bib-0030] Hamaguchi, M. , H. Shimabukuro , M. Kawane , and T. Hamaguchi . 2013. “A New Record of the Kumamoto Oyster *Crassostrea sikamea* in the Seto Inland Sea, Japan.” Marine Biodiversity Records 6: e16. 10.1017/S1755267212001297.

[eva70100-bib-0031] Heare, J. E. , B. Blake , J. P. Davis , B. Vadopalas , and S. B. Roberts . 2017. “Evidence of *Ostrea lurida* Carpenter, 1864, Population Structure in Puget Sound, WA, USA.” Marine Ecology 38: e12458.

[eva70100-bib-0032] Hedgecock, D. , M. Banks , and D. McGoldrick . 1993. “The Status of the Kumamoto Oyster *Crassostrea sikamea* (Amemiya 1928) in U.S. Commercial Brood Stocks.” Journal of Shellfish Research 12: 215–221.

[eva70100-bib-0033] Hedgecock, D. , G. Li , M. Banks , and Z. Kain . 1999. “Occurrence of the Kumamoto Oyster *Crassostrea sikamea* in the Ariake Sea, Japan.” Marine Biology 133: 65–68.

[eva70100-bib-0034] Hu, B. , Y. Tian , Q. Li , and S. Liu . 2022. “Genomic Signatures of Artificial Selection in the Pacific Oyster, *Crassostrea gigas* .” Evolutionary Applications 15: 618–630. 35505882 10.1111/eva.13286 PMC9046764

[eva70100-bib-0035] Hu, L. , Z. Zhang , H. Wang , and T. Zhang . 2018. “Molecular Phylogeography and Population History of *Crassostrea sikamea* (Amemiya, 1928) Based on Mitochondrial DNA.” Journal of Experimental Marine Biology and Ecology 503: 23–30.

[eva70100-bib-0036] Hu, L.‐S. , and Y.‐W. Dong . 2022. “Multiple Genetic Sources Facilitate the Northward Range Expansion of an Intertidal Oyster Along China's Coast.” Ecological Applications 34: e2764. 36259430 10.1002/eap.2764

[eva70100-bib-0037] In, V. , W. O'Connor , V. Sang , P. Van , and W. Knibb . 2017. “Resolution of the Controversial Relationship Between Pacific and Portuguese Oysters Internationally and in Vietnam.” Aquaculture 473: 389–399. 10.1016/j.aquaculture.2017.03.004.

[eva70100-bib-0205] Jiao, Z. , Y. Tian , B. Hu , Q. Li , and S. Liu . 2021. “Genome Structural Variation Landscape and its Selection Signatures in the Fast‐growing Strains of the Pacific Oyster, *Crassostrea gigas* .” Marine Biotechnology 23: 736–748. 34498173 10.1007/s10126-021-10060-5

[eva70100-bib-0038] Kanehisa, M. , S. Goto , Y. Sato , M. Furumichi , and M. Tanabe . 2012. “KEGG for Integration and Interpretation of Large‐Scale Molecular Data Sets.” Nucleic Acids Research 40: D109–D114. 22080510 10.1093/nar/gkr988 PMC3245020

[eva70100-bib-0206] Kenny, N. J. , S. A. McCarthy , O. Dudchenko , et al. 2020. “The Gene‐Rich Genome of the Scallop.” GigaScience 9: giaa037. 32352532 10.1093/gigascience/giaa037 PMC7191990

[eva70100-bib-0039] Khan, P. , B. Linkhart , and H.‐G. Simon . 2002. “Different Regulation of T‐Box Genes Tbx4 and Tbx5 During Limb Development and Limb Regeneration.” Developmental Biology 250: 383–392. 12376111

[eva70100-bib-0040] Koren, S. , B. P. Walenz , K. Berlin , J. R. Miller , N. H. Bergman , and A. M. Phillippy . 2017. “Canu: Scalable and Accurate Long‐Read Assembly via Adaptive k‐Mer Weighting and Repeat Separation.” Genome Research 27: 722–736. 28298431 10.1101/gr.215087.116 PMC5411767

[eva70100-bib-0041] Kumar, S. , M. Suleski , J. M. Craig , et al. 2022. “TimeTree 5: An Expanded Resource for Species Divergence Times.” Molecular Biology and Evolution 39, no. 8: msac174. 10.1093/molbev/msac174. 35932227 PMC9400175

[eva70100-bib-0042] Li, A. , H. Dai , X. Guo , et al. 2021. “Genome of the Estuarine Oyster Provides Insights Into Climate Impact and Adaptive Plasticity.” Communications Biology 4: 1287. 34773106 10.1038/s42003-021-02823-6 PMC8590024

[eva70100-bib-0043] Li, C. , Q. Kou , Z. Zhang , et al. 2021. “Reconstruction of the Evolutionary Biogeography Reveal the Origins and Diversification of Oysters (Bivalvia: Ostreidae).” Molecular Phylogenetics and Evolution 164: 107268. 10.1016/j.ympev.2021.107268. 34302948

[eva70100-bib-0044] Li, H. , and R. Durbin . 2011. “Inference of Human Population History From Individual Whole‐Genome Sequences.” Nature 475: 493–496. 21753753 10.1038/nature10231 PMC3154645

[eva70100-bib-0045] Li, H. , B. Handsaker , A. Wysoker , et al. 2009. “The Sequence Alignment/Map Format and SAMtools.” Bioinformatics 25: 2078–2079. 19505943 10.1093/bioinformatics/btp352 PMC2723002

[eva70100-bib-0046] Li, H. , Q. Li , H. Yu , and S. Du . 2019. “Developmental Dynamics of Myogenesis in Pacific Oyster *Crassostrea gigas* .” Comparative Biochemistry and Physiology. Part B, Biochemistry & Molecular Biology 227: 21–30. 10.1016/j.cbpb.2018.08.008 30193833

[eva70100-bib-0047] Li, H. , H. Yu , and Q. Li . 2021. “Striated Myosin Heavy Chain Gene Is a Crucial Regulator of Larval Myogenesis in the Pacific Oyster *Crassostrea gigas* .” International Journal of Biological Macromolecules 179: 388–397. 33689771 10.1016/j.ijbiomac.2021.03.022

[eva70100-bib-0048] Li, L. , J. X. Huang , and Y. H. Liu . 2024. “The Extracellular Matrix Glycoprotein Fibrillin‐1 in Health and Disease.” Frontiers in Cell and Developmental Biology 11: 1302285. 38269088 10.3389/fcell.2023.1302285 PMC10806136

[eva70100-bib-0049] Li, M. , S. Tian , L. Jin , et al. 2013. “Genomic Analyses Identify Distinct Patterns of Selection in Domesticated Pigs and Tibetan Wild Boars.” Nature Genetics 45: 1431–1438. 24162736 10.1038/ng.2811

[eva70100-bib-0050] Lin, Q. , S. Fan , Y. Zhang , et al. 2016. “The Seahorse Genome and the Evolution of Its Specialized Morphology.” Nature 540: 395–399. 27974754 10.1038/nature20595 PMC8127814

[eva70100-bib-0051] Ma, H. T. , W. G. Lv , Y. P. Qin , et al. 2022. “Aquaculture Potential of Two Kumamoto Oyster (*Crassostrea sikamea*) Populations and Their Reciprocal Hybrids in Southern China.” Aquaculture 546: 737301. 10.1016/j.aquaculture.2021.737301.

[eva70100-bib-0052] Marcais, G. , and C. Kingsford . 2011. “A Fast, Lock‐Free Approach for Efficient Parallel Counting of Occurrences of k‐Mer.” Bioinformatics 27: 764–770. 21217122 10.1093/bioinformatics/btr011 PMC3051319

[eva70100-bib-0053] O'Brien, S. J. , M. Menotti‐Raymond , W. J. Murphy , et al. 1999. “The Promise of Comparative Genomics in Mammals.” Science 286: 458–462, 479–481. 10521336 10.1126/science.286.5439.458

[eva70100-bib-0054] Penaloza, C. , A. P. Gutierrez , L. Eory , et al. 2021. “A Chromosome‐Level Genome Assembly for the Pacific Oyster *Crassostrea gigas* .” GigaScience 10: giab020. 33764468 10.1093/gigascience/giab020 PMC7992393

[eva70100-bib-0055] Powell, D. , S. Subramanian , S. Suwansa‐ard , et al. 2018. “The Genome of the Oyster Saccostrea Offers Insight Into the Environmental Resilience of Bivalves.” DNA Research 25: 655–665. 30295708 10.1093/dnares/dsy032 PMC6289776

[eva70100-bib-0056] Powell, S. , D. Szklarczyk , K. Trachana , et al. 2012. “eggNOG v3.0: Orthologous Groups Covering 1133 Organisms at 41 Different Taxonomic Ranges.” Nucleic Acids Research 40: D284–D289. 22096231 10.1093/nar/gkr1060 PMC3245133

[eva70100-bib-0057] Puritz, J. , H. Zhao , X. Guo , et al. 2022. “Nucleotide and Structural Polymorphisms of the Eastern Oyster Genome Paint a Mosaic of Divergence, Selection, and Human Impacts.” *bioRxiv* 2022.08.29.505629. 10.1101/2022.08.29.505629.

[eva70100-bib-0058] Puritz, J. B. , X. Guo , M. Hare , et al. 2023. “A Second Unveiling: Haplotig Masking of the Eastern Oyster Genome Improves Population‐Level Inference.” Molecular Ecology Resources 24: e13801. 37186213 10.1111/1755-0998.13801

[eva70100-bib-0059] Qi, H. , L. Li , and G. Zhang . 2021. “Construction of a Chromosome‐Level Genome and Variation Map for the Pacific Oyster *Crassostrea gigas* .” Molecular Ecology Resources 21: 1670–1685. 33655634 10.1111/1755-0998.13368

[eva70100-bib-0060] Qumsiyeh, M. B. 1994. “Evolution of Number and Morphology of Mammalian Chromosomes.” Journal of Heredity 85: 455–465. 7995926 10.1093/oxfordjournals.jhered.a111501

[eva70100-bib-0061] Qumsiyeh, M. B. , and E. N. Handal . 2022. “Adaptive Nature of Chromosome Variation in Placental Mammals and Applicability to Domestication and Invasiveness.” Hystrix‐Italian Journal of Mammalogy 33: 102–106.

[eva70100-bib-0062] Roberts, A. , H. Pimentel , C. Trapnell , and L. Pachter . 2011. “Identification of Novel Transcripts in Annotated Genomes Using RNA‐Seq.” Bioinformatics 27: 2325–2329. 21697122 10.1093/bioinformatics/btr355

[eva70100-bib-0063] Sekino, M. 2009. “In Search of the Kumamoto Oyster *Crassostrea Sikamea* (Amemiya, 1928) Based on Molecular Markers: Is the Natural Resource at Stake?” Fisheries Science 75: 819–831.

[eva70100-bib-0064] Sekino, M. , S. i. Sato , J.‐S. Hong , and Q. Li . 2012. “Contrasting Pattern of Mitochondrial Population Diversity Between an Estuarine Bivalve, the Kumamoto Oyster *Crassostrea sikamea*, and the Closely Related Pacific Oyster *C. gigas* .” Marine Biology 159: 2757–2776. 10.1007/s00227-012-2037-z.

[eva70100-bib-0065] Smaldone, S. , C. L. Bigarella , M. del Solar , S. Ghaffari , and F. Ramirez . 2016. “Fibrillin‐1 Microfibrils Influence Adult Bone Marrow Hematopoiesis.” Matrix Biology 52–54: 88–94. 10.1016/j.matbio.2015.11.006 PMC4875809 26610678

[eva70100-bib-0066] Song, H. , X. Guo , L. Sun , et al. 2021. “The Hard Clam Genome Reveals Massive Expansion and Diversification of Inhibitors of Apoptosis in Bivalvia.” BMC Biology 19: 15. 33487168 10.1186/s12915-020-00943-9 PMC7831173

[eva70100-bib-0067] Stanke, M. , R. Steinkamp , S. Waack , and B. Morgenstern . 2004. “AUGUSTUS: A Web Server for Gene Finding in Eukaryotes.” Nucleic Acids Research 32: W309–W312. 15215400 10.1093/nar/gkh379 PMC441517

[eva70100-bib-0068] Sun, J. , R. Li , C. Chen , J. D. Sigwart , and K. M. Kocot . 2021. “Benchmarking Oxford Nanopore Read Assemblers for High‐Quality Molluscan Genomes.” Philosophical Transactions of the Royal Society, B: Biological Sciences 376: 20200160. 10.1098/rstb.2020.0160 PMC8059532 33813888

[eva70100-bib-0069] Sutherland, B. J. G. , N. Itoh , K. Gilchrist , B. Boyle , M. Roth , and T. J. Green . 2023. “Genomic Diversity of Wild and Cultured Yesso Scallop Mizuhopecten Yessoensis From Japan and Canada.” G3: Genes, Genomes, Genetics 13, no. 12: jkad242. 10.1093/g3journal/jkad242. 37857308 PMC10700054

[eva70100-bib-0070] Sutherland, B. J. G. , C. Rycroft , A. L. Ferchaud , et al. 2020. “Relative Genomic Impacts of Translocation History, Hatchery Practices, and Farm Selection in Pacific Oyster *Crassostrea gigas* Throughout the Northern Hemisphere.” Evolutionary Applications 13: 1380–1399. 32684965 10.1111/eva.12965 PMC7359842

[eva70100-bib-0207] Tarailo‐Graovac, M. , and N. Chen . 2009. “Using RepeatMasker to Identify Repetitive Elements in Genomic Sequences.” Current Protocols in Bioinformatics, Chapter 4: 4.10.1–4.10.14. 10.1002/0471250953.bi0410s25 19274634

[eva70100-bib-0071] Teng, W. , H. Fu , Z. Li , et al. 2023. “Parallel Evolution in Crassostrea Oysters Along the Latitudinal Gradient Is Associated With Variation in Multiple Genes Involved in Adipogenesis.” Molecular Ecology 32: 5276–5287. 37606178 10.1111/mec.17108

[eva70100-bib-0072] Ter‐Hovhannisyan, V. , A. Lomsadze , Y. O. Chernoff , and M. Borodovsky . 2008. “Gene Prediction in Novel Fungal Genomes Using an Ab Initio Algorithm With Unsupervised Training.” Genome Research 18: 1979–1990. 18757608 10.1101/gr.081612.108 PMC2593577

[eva70100-bib-0073] Wang, H. , and X. Guo . 2008. “Identification of Crassostrea Ariakensis and Related Oysters by Multiplex Species‐Specific PCR.” Journal of Shellfish Research 27: 481–487.

[eva70100-bib-0074] Wang, H. , J. Lv , Q. Zeng , et al. 2021. “Genetic Differentiation and Selection Signatures in Two Bay Scallop ( *Argopecten irradians* ) Breeds Revealed by Whole‐Genome Resequencing Analysis.” Aquaculture 543: 736944.

[eva70100-bib-0075] Wang, H. , L. Qian , A. Wang , and X. Guo . 2013. “Occurrence and Distribution of *Crassostrea sikamea* (Amemiya 1928) in China.” Journal of Shellfish Research 32: 439–446.

[eva70100-bib-0076] Wang, K. , M. Li , and H. Hakonarson . 2010. “ANNOVAR: Functional Annotation of Genetic Variants From High‐Throughput Sequencing Data.” Nucleic Acids Research 38: e164. 20601685 10.1093/nar/gkq603 PMC2938201

[eva70100-bib-0077] Wang, S. , J. Zhang , W. Jiao , et al. 2017. “Scallop Genome Provides Insights Into Evolution of Bilaterian Karyotype and Development.” Nature Ecology & Evolution 1: 120. 28812685 10.1038/s41559-017-0120 PMC10970998

[eva70100-bib-0078] Wang, X. , Z. Yang , L. Jiang , et al. 2023. “Assessment of Germplasm Resource and Detection of Genomic Signature Under Artificial Selection of Zhikong Scallop (*Chlamys farreri*).” Aquaculture 574: 739730.

[eva70100-bib-0079] Wang, Y. , H. Tang , J. D. DeBarry , et al. 2012. “MCScanX: A Toolkit for Detection and Evolutionary Analysis of Gene Synteny and Collinearity.” Nucleic Acids Research 40: e49. 10.1093/nar/gkr1293. 22217600 PMC3326336

[eva70100-bib-0080] Wang, Y. , Z. Xu , and X. Guo . 2004. “Differences in the rDNA‐Bearing Chromosome Divide the Asian‐Pacific and Atlantic Species of Crassostrea (Bivalvia, Mollusca).” Biological Bulletin 206: 46–54. 14977729 10.2307/1543197

[eva70100-bib-0081] Wang, Z. , S. Casas , J. La Peyre , et al. 2025. “Genomic Selection for Dermo Resistance in the Eastern Oyster *Crassostrea virginica*: Production and Laboratory Testing of F1 Generation.” Journal of Shellfish Research 44, no. 1: 1–13.

[eva70100-bib-0082] Wu, B. , X. Chen , M. Yu , et al. 2021. “Chromosome‐Level Genome and Population Genomic Analysis Provide Insights Into the Evolution and Environmental Adaptation of Jinjiang Oyster *Crassostrea ariakensis* .” Molecular Ecology Resources 22, no. 4: 1529–1544. 34800349 10.1111/1755-0998.13556

[eva70100-bib-0083] Xu, H. , L. Kong , H. Yu , and S. Liu . 2019. “Fertilization, Survival and Growth of Hybrids Between *Crassostrea gigas* and *Crassostrea sikamea* .” Fisheries Science 85: 821–828.

[eva70100-bib-0209] Xuan, Y. , G. Chang , S. Liu , Z. Lin , and Q. Xue . 2024. “A Comparison of the Mitochondrial DNA‐Based Genetic Diversity of Kumamoto Oyster Populations From China, Japan, and the United States.” Marine Sciences 48, no. 9: 24–33. (In Chinese with a English abstract).

[eva70100-bib-0084] Yang, J. A. , S. H. Lee , M. E. Goddard , and P. M. Visscher . 2011. “GCTA: A Tool for Genome‐Wide Complex Trait Analysis.” American Journal of Human Genetics 88: 76–82. 21167468 10.1016/j.ajhg.2010.11.011 PMC3014363

[eva70100-bib-0208] Yang, J.‐L. , D.‐D. Feng , J. Liu , et al. 2021. “Chromosome‐Level Genome Assembly of the Hard‐Shelled Mussel *Mytilus coruscus*, a Widely Distributed Species From the Temperate Areas of East Asia.” GigaScience 10: giab024. 33891010 10.1093/gigascience/giab024 PMC8063583

[eva70100-bib-0085] Yang, Z. 2007. “PAML 4: Phylogenetic Analysis by Maximum Likelihood.” Molecular Biology and Evolution 24: 1586–1591. 17483113 10.1093/molbev/msm088

[eva70100-bib-0086] Zeng, D. , and X. Guo . 2022. “Mantle Transcriptome Provides Insights Into Biomineralization and Growth Regulation in the Eastern Oyster ( *Crassostrea virginica* ).” Marine Biotechnology 24: 82–96. 34989931 10.1007/s10126-021-10088-7

[eva70100-bib-0087] Zhang, C. , S. S. Dong , J. Y. Xu , W. M. He , and T. L. Yang . 2019. “PopLDdecay: A Fast and Effective Tool for Linkage Disequilibrium Decay Analysis Based on Variant Call Format Files.” Bioinformatics 35: 1786–1788. 30321304 10.1093/bioinformatics/bty875

[eva70100-bib-0088] Zhang, G. , X. Fang , X. Guo , et al. 2012. “The Oyster Genome Reveals Stress Adaptation and Complexity of Shell Formation.” Nature 490: 49–54. 22992520 10.1038/nature11413

[eva70100-bib-0089] Zhang, L. , L. Li , X. Guo , G. W. Litman , L. J. Dishaw , and G. Zhang . 2015. “Massive Expansion and Functional Divergence of Innate Immune Genes in a Protostome.” Scientific Reports 5: 8693. 25732911 10.1038/srep08693 PMC4346834

[eva70100-bib-0090] Zhang, Y. , F. Mao , S. Xiao , et al. 2022. “Comparative Genomics Reveals Evolutionary Drivers of Sessile Life and Left‐Right Shell Asymmetry in Bivalves.” Genomics, Proteomics & Bioinformatics 20: 1078–1091. 10.1016/j.gpb.2021.10.005 PMC10225672 35091095

[eva70100-bib-0091] Zhao, H. , X. Guo , W. Wang , et al. 2024. “Consequences of Domestication in Eastern Oyster: Insights From Whole Genomic Analyses.” Evolutionary Applications 17: e13710. 38817396 10.1111/eva.13710 PMC11134191

[eva70100-bib-0092] Zheng, J. , X. Wang , T. Feng , et al. 2023. “Molecular Mechanisms Underlying Hematophagia Revealed by Comparative Analyses of Leech Genomes.” GigaScience 12: giad023. 10.1093/gigascience/giad023 PMC10087013 37039117

