# Supplementary material for: Genome of Kumamoto Oyster Crassostrea sikamea Provides Insights Into Bivalve Evolution and Environmental Adaptation
Source: Evol Appl. 2025 Apr 24;18(4):e70100. doi: 10.1111/eva.70100 (PMC12021676; doi:10.1111/eva.70100)
Supplement: Supplementary file 1 — Figure S1 Seawater temperature in seven populations/subpopulations. Asia locations were shown with satellite data (2010–2019) and US location with data during Sep, 2013 to Aug, 2014 in Oyster Bay which is near to the US population cultured region, the Dabob bay, from reference (Heare 2017). Figure S2 Genome size estimation based on the k‐mer, according to the genome survey results, Depth = 109 is the main peak, and the genome size was calculated by the formula (Genome size = Kmer‐number/depth) is about 523,972,884 bp. Depth = 54 (approximately 1/2 of the main peak position) and Depth196 are considered to be caused by genome duplication. Figure S3 Heatmap of genome‐wide all‐by‐all Hi‐C interaction of all the chromosomes, the horizontal and vertical coordinates represent chromosomes, and the darker the color in the diagram, the stronger the interaction signal. Figure S4 Genome assessment based on GC content, GC Depth, mapping rate and coverage. The figure shows the distribution of GC in genome assembly sequences. The horizontal axis represents the GC content, which is calculated as a 10 kb window. The vertical axis represents the proportion of the number of windows corresponding to the GC content to the total number of windows (A). The figure shows the GC Depth scatter plot, with GC content on the horizontal axis and Depth on the vertical axis. These two values are calculated in sequence using a 10 Kb window. There is no obvious left and right block phenomenon in the GC Depth diagram, indicating no contamination and high assembly quality (B). The mapping rate, coverage, and depth distribution after genome assembly sequence alignment, with the horizontal axis representing the sequencing depth value, which is calculated in sequence using a 10 Kb window(C). Figure S5 Comparison of gene structure including gene length distribution (1), exon length distribution (2), CDS length distribution (3), intron length distribution (4), and exon number distribution (5) among related [file EVA-18-e70100-s002.pdf]

Genome of Kumamoto oyster *Crassostrea sikamea* provides insights into bivalve evolution and environmental adaptation

Sheng Liu[1,2#], Youli Liu[1,2#], Ximing Guo[3#], Naoki Itoh[4], Guangqiu Chang[1,2], Zhihua Lin[1,2] and Qinggang Xue[1,2*]

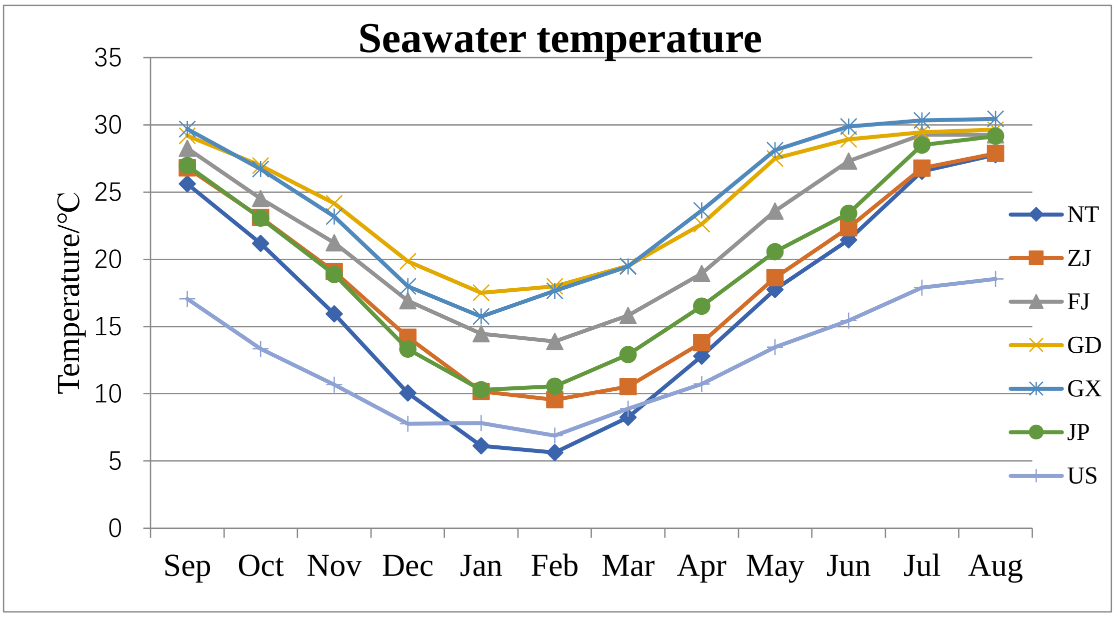

Figure S1 Seawater temperature in seven populations/sub-populations. Asia locations were shown with satellite data (2010 to 2019) and US location with data during Sep, 2013 to Aug, 2014 in Oyster Bay which is near to the US population cultured region, the Dabob bay, from reference (Heare 2017).

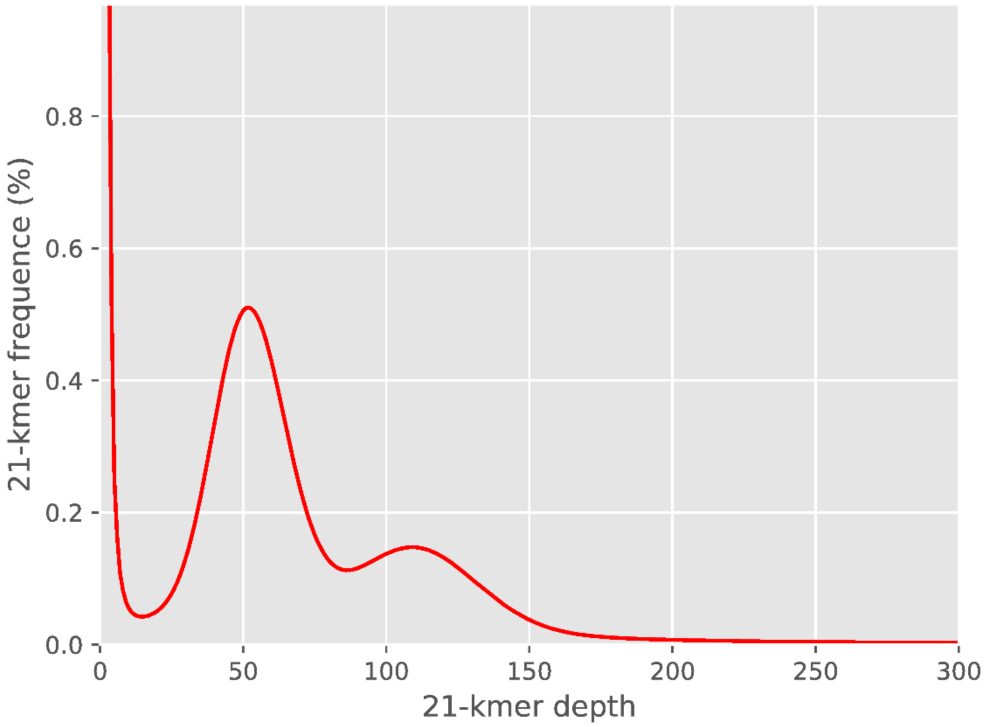

Figure S2 Genome size estimation based on the k-mer, according to the genome survey results, Depth=109 is the main peak, and the genome size was calculated by the formula (Genome size=Kmer-number/depth) is about 523,972,884 bp. Depth=54 (approximately 1/2 of the main peak position) and Depth<54 ahead of the main peak position are considered to be caused by genome heterozygosity, and after the main peak position Depth=196 (approximately twice the main peak position) and Depth>196 are considered to be caused by genome duplication.

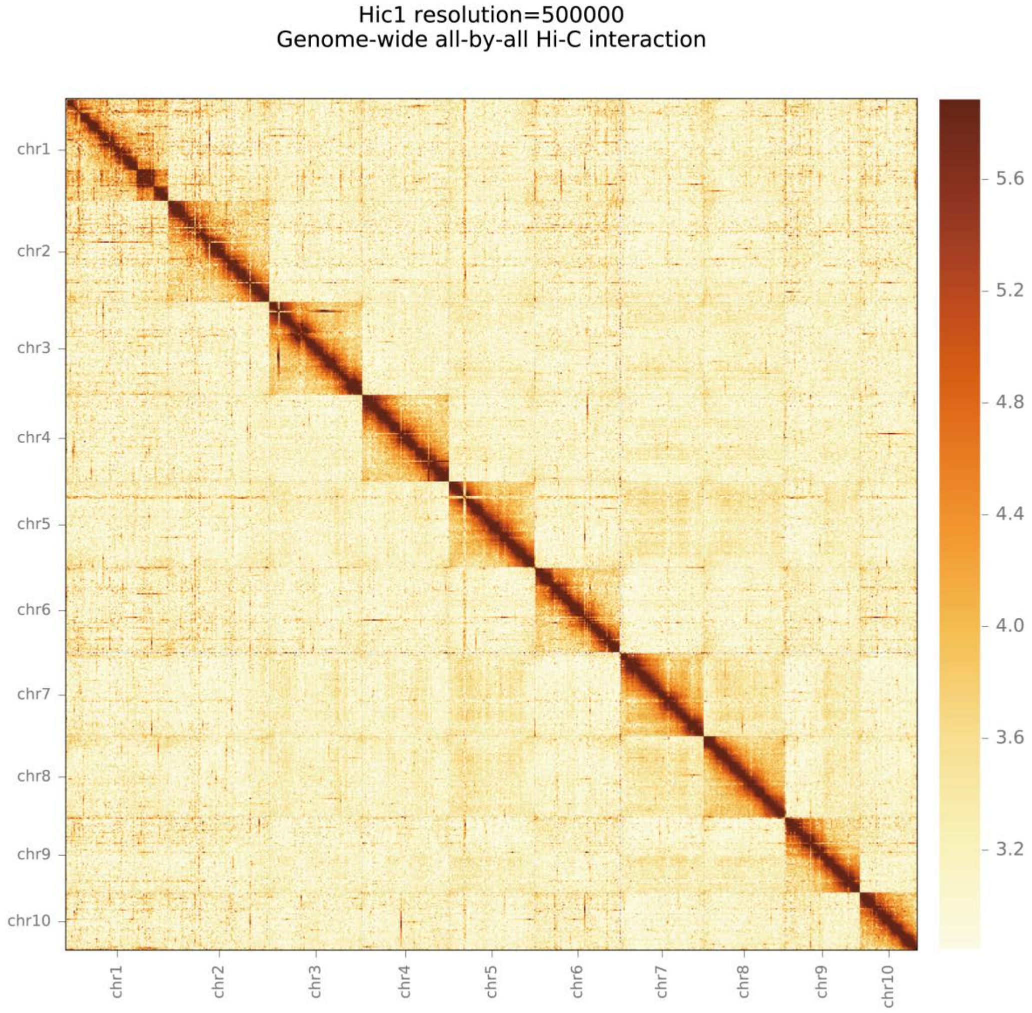

Figure S3 Heatmap of genome-wide all-by-all Hi-C interaction of all the chromosomes, the horizontal and vertical coordinates represent chromosomes, and the darker the color in the diagram, the stronger the interaction signal.

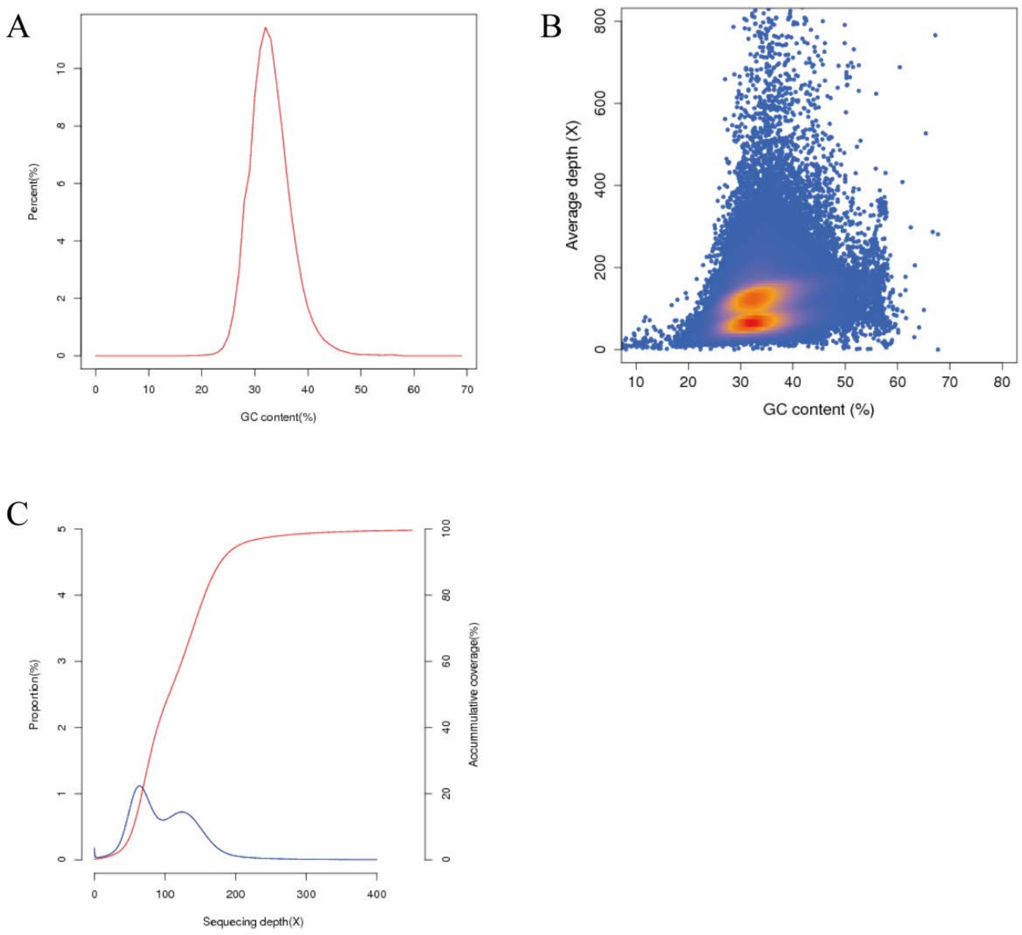

Figure S4 Genome assessment based on GC content, GC Depth, mapping rate and coverage. The figure shows the distribution of GC in genome assembly sequences. The horizontal axis represents the GC content, which is calculated as a 10 kb window. The vertical axis represents the proportion of the number of windows corresponding to the GC content to the total number of windows (A). The figure shows the GC Depth scatter plot, with GC content on the horizontal axis and Depth on the vertical axis. These two values are calculated in sequence using a 10 Kb window. There is no obvious left and right block phenomenon in the GC Depth diagram, indicating no contamination and high assembly quality (B). The mapping rate, coverage, and depth distribution after genome assembly sequence alignment, with the horizontal axis representing the sequencing depth value, which is calculated in sequence using a 10 Kb window(C).

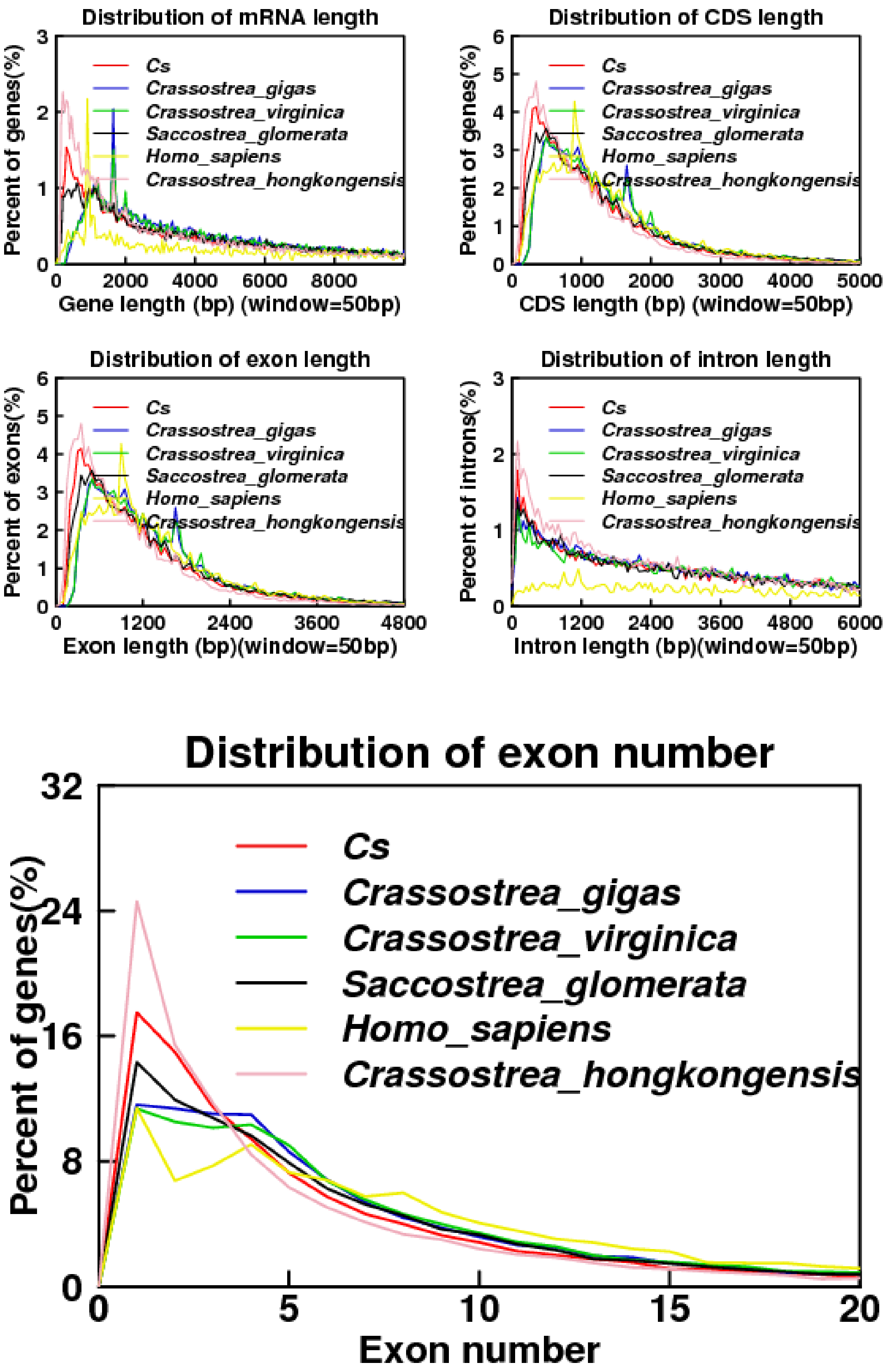

Figure S5 Comparison of gene structure including gene length distribution (1), exon length distribution (2), CDS length distribution (3), intron length distribution (4), and exon number

distribution (5) among related species (Cs referred to *Crassostrea sikamea*).

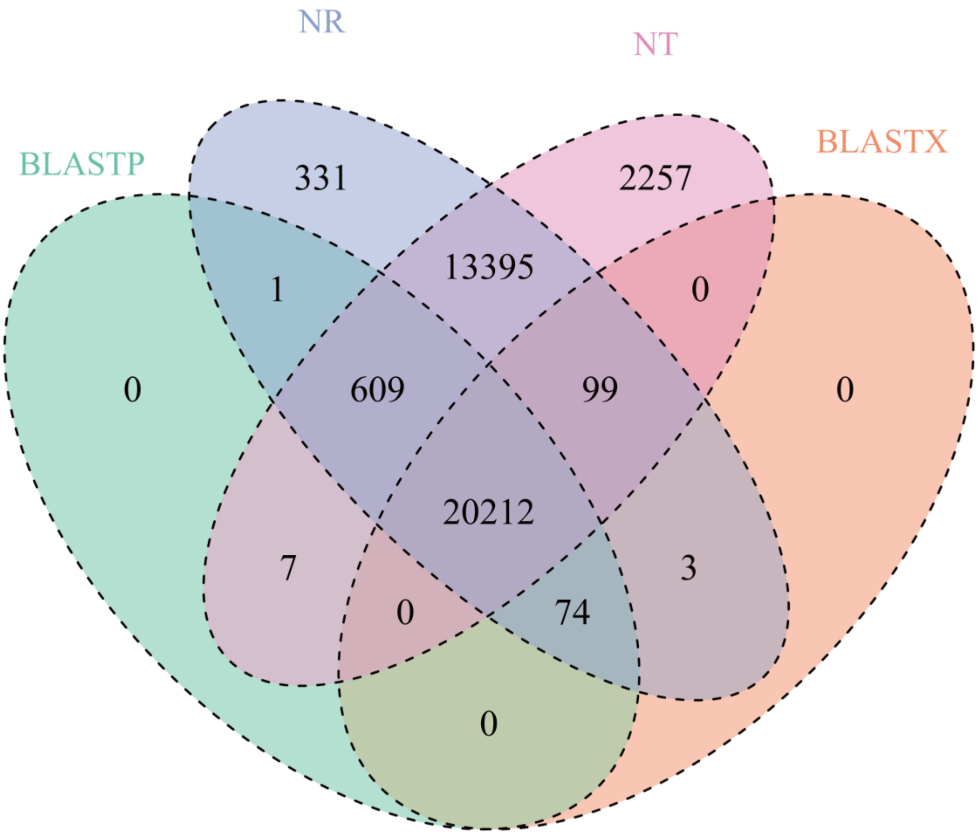

Figure S6 Venn map for comparison of annotated genes in NT, NR, Uniprot-BLASTX, and Uniprot-BLASTP databases in NCBI.

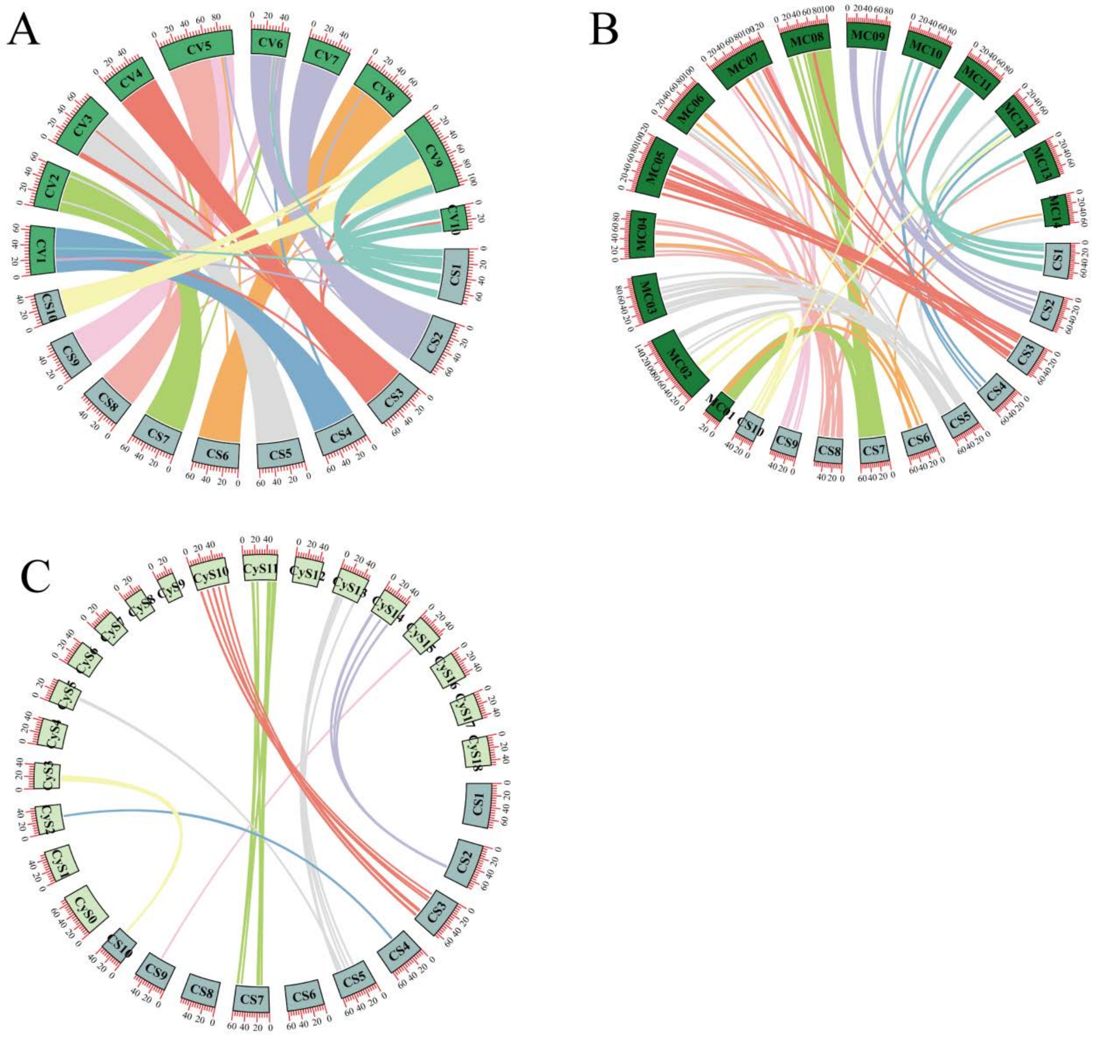

Figure S7 Chromosome synteny between *C. sikamea* and oyster *C. virginica* (A), mussel *M. coruscus* (n=14) (B), and clam *C. sinensis* (n=19) (C).

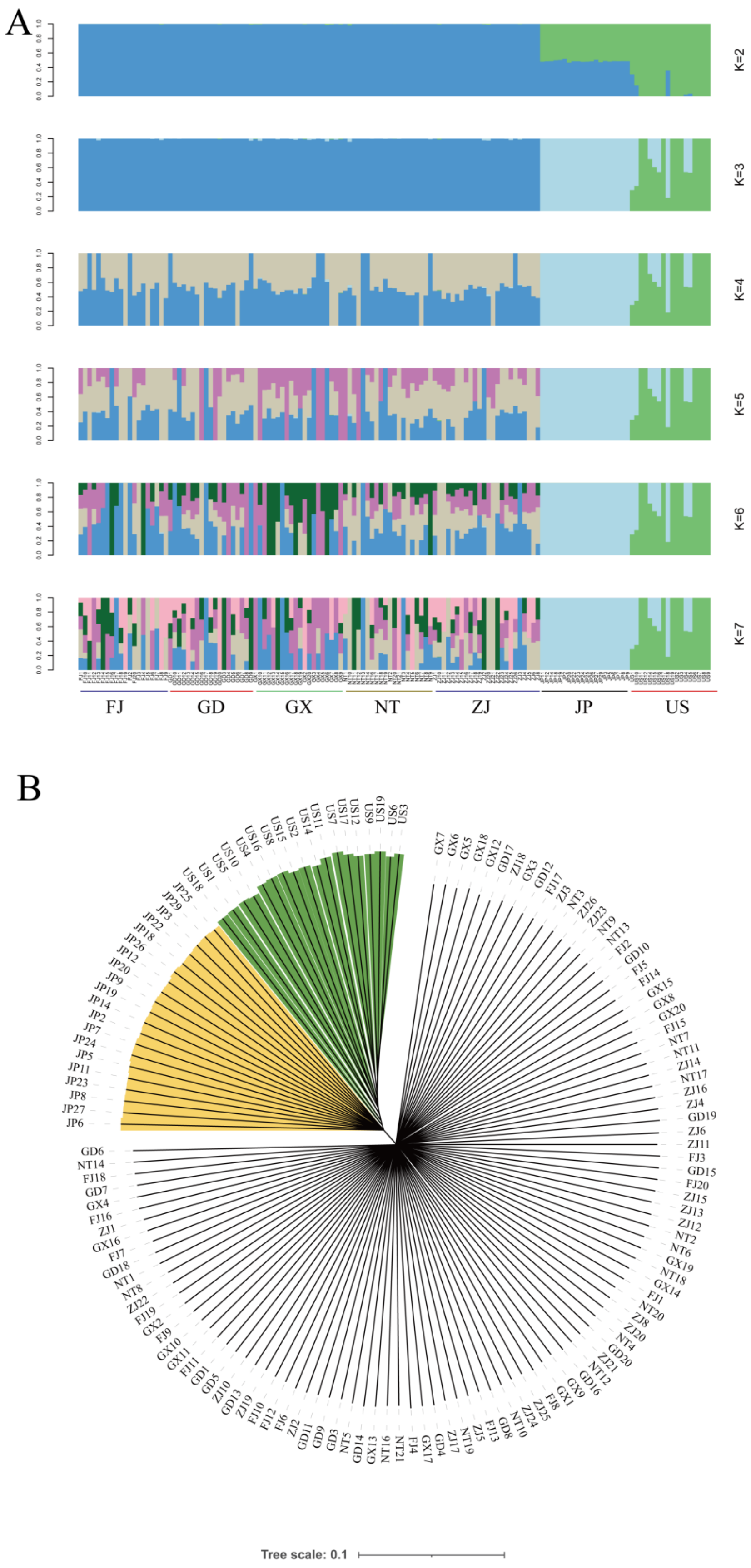

Figure S8 Genetic admixture structure (A) and phylogenetic tree (B) for 141 re-sequencing individuals in seven populations. These results was inferred using the 1,954,312 high quality filtered

SNPs, yellow and green color represent individuals from Japan and US, respectively in (B).

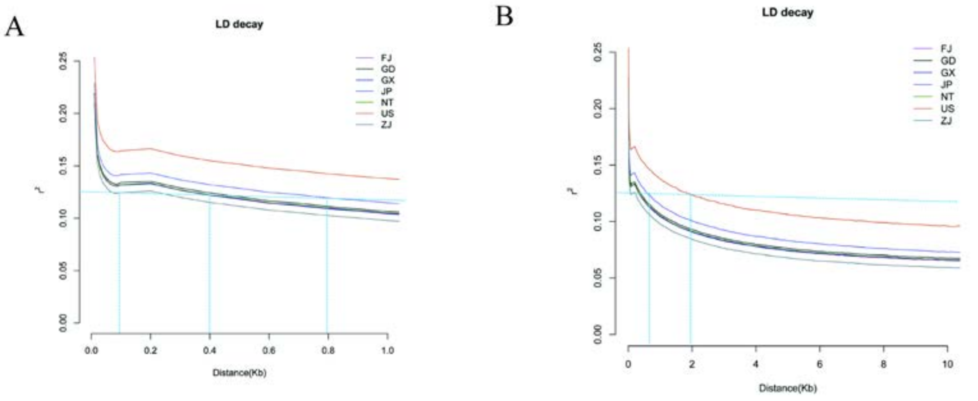

Figure S9: Linkage disequilibrium (LD) decay for seven populations/sub-populations. The horizontal axis represents the distance at which LD occurs, while the vertical axis represents the correlation coefficient of LD (r^2), vertical dashed line represent corresponding distance for LD decay to half of its maximum valve.

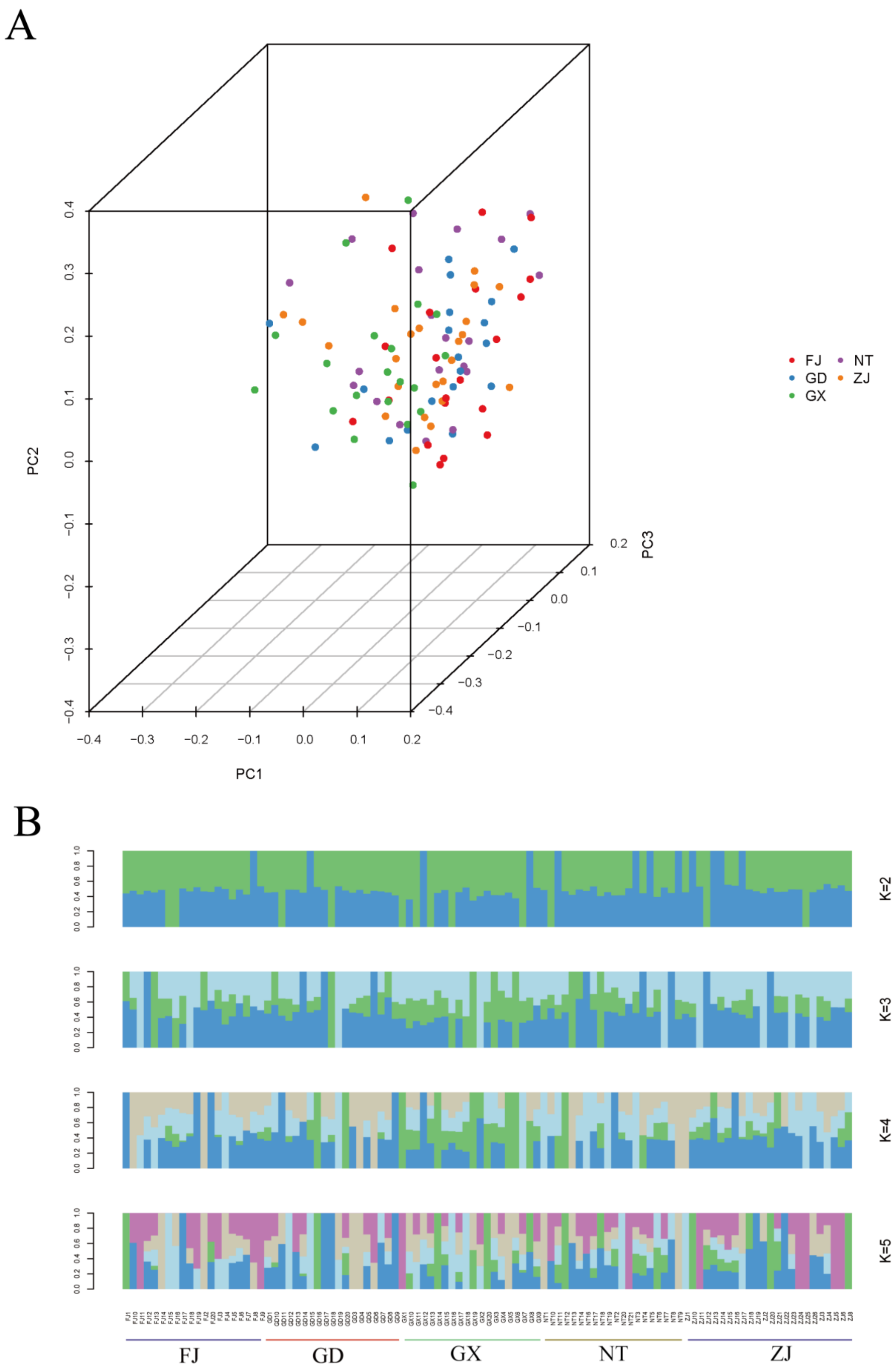

Figure S10 Population structure analysis for five Chinese populations, PCA (A), and population structure (B).

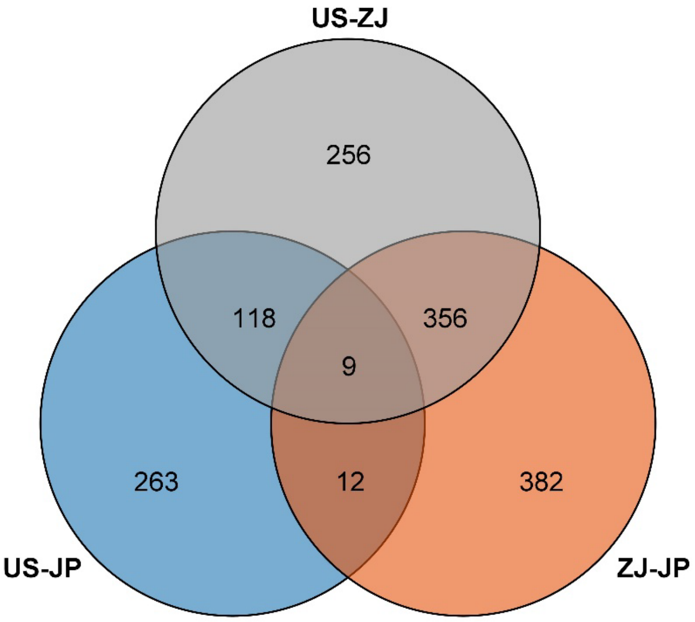

Figure S11 Venn map for shared genes between ZJ with JP, US with JP and US with ZJ.
