# Supplementary material for: Genome of Kumamoto Oyster Crassostrea sikamea Provides Insights Into Bivalve Evolution and Environmental Adaptation
Source: Evol Appl. 2025 Apr 24;18(4):e70100. doi: 10.1111/eva.70100 (PMC12021676; doi:10.1111/eva.70100)
Supplement: Supplementary file 2 — Table S1 Fifteen molluscan species used for phylogenetic analysis and sources for genome data. Table S2 Location information for C. sikamea populations sampled from China, Japan and the US. Table S3 Summary statistics of sequencing data used for genome assembly annotation. Table S4 K‐mer statistics from C. sikamea genome survey analysis. Table S5 Statistics for contig level assembly of C. sikamea genome. Table S6 Statistics for chromosome level assembly of C. sikamea genome. Table S7 Mapping and coverage rate based on whole‐genome short sequence reads. Table S8 Repeat content in C. sikamea genome predicted with different methods. Table S9 Types and proportion of different transposable elements in C. sikamea genome. Table S10 Types and proportion of noncoding RNA in C. sikamea genome. Table S11 Statistics for gene prediction by different methods. Table S12 Statistics for C. sikamea genes predicted with different organisms based on homologous genes in related species. Table S13 Gene homologues annotated in different databases. Table S14 Mapping rate and average depth of resequencing data from different populations. Table S15 Genome‐wide SNP annotation and statistics after filtering. Table S16 Population fixation index (F ST) data for seven populations based on genome‐wide SNPs. Table S17 Blocks showing selection sweep signals and associated genes summary from different comparisons. [file EVA-18-e70100-s001.docx]

**Genome of Kumamoto oyster *Crassostrea sikamea* provides insights into bivalve evolution and environmental adaptation**

**Sheng Liu^1,2#^, Youli Liu^1,2#^, Ximing Guo^3#^, Naoki Itoh^4^, Guangqiu Chang^1,2^, Zhihua Lin^1,2^ and Qinggang Xue^1,2*^**

Table S1 Fifteen molluscan species used for phylogenetic analysis and sources for genome data.

| Name | Abbr. | Class | Order | Source |
| --- | --- | --- | --- | --- |
| *Crassostrea sikamea* | *C.sikamea* | Bivalvia | Ostreida | This study |
| *Crassostrea gigas* | *C.gigas* | Bivalvia | Ostreida | <ftp://ftp.ncbi.nlm.nih.gov/genomes/all/GCA/011/032/805/GCA_011032805.1_ASM1103280v1> |
| *Crassostrea angulata* | *C. angulata* | Bivalvia | Ostreida | <https://www.ncbi.nlm.nih.gov/datasets/genome/GCA_025765675.3/> |
| *Crassostrea hongkongensis* | *C.hongkongensis* | Bivalvia | Ostreida | <https://ngdc.cncb.ac.cn/gsa> |
| *Crassostrea virginica* | *C.virginica* | Bivalvia | Ostreida | <https://www.ncbi.nlm.nih.gov/assembly/GCF_002022765.2/> |
| *Saccostrea glomerata* | *S.glomerata* | Bivalvia | Ostreida | <http://soft.bioinfo-minzhao.org/srog/> |
| *Pinctada fucata* | *P.fucata* | Bivalvia | Ostreida | <https://marinegenomics.oist.jp/pearl_4_1B/viewer/download?project_id=111> |
| *Mercenaria mercenaria* | *M.mercenaria* | Bivalvia | Venerida | <https://figshare.com/s/a8378910b437fc843a46> |
| *Cyclina sinensis* | *C.sinensis* | Bivalvia | Venerida | <https://doi.org/10.5061/dryad.44j0zpcb5> |
| *Sinonovacula constricta* | *S.constricta* | Bivalvia | Solenida | <https://www.ncbi.nlm.nih.gov/assembly/GCA_009762815.1> |
| *Pecten maximus* | *P. maximus* | Bivalvia | Pectinida | <https://figshare.com/articles/dataset/Pecten_maximus_genome_gene_models_annotations_and_related_files/10311068> |
| *Mizuhopecten yessoensis* | *M.yessoensis* | Bivalvia | Pectinida | <https://www.ncbi.nlm.nih.gov/assembly/GCF_002113885.1> |
| *Octopus bimaculoides* | *O.bimaculoides* | Cephalopoda | Octopoda | <https://ftp.ncbi.nlm.nih.gov/genomes/all/GCF/001/194/135/GCF_001194135.1_Octopus_bimaculoides_v2_0> |
| *Aplysia californica* | *A.californica* | Gastropoda | Aplysiida | <https://ftp.ncbi.nlm.nih.gov/genomes/all/GCF/000/002/075/GCF_000002075.1_AplCal3.0/> |
| *Lottia gigantea* | *L.gigantea* | Gastropoda | Lottioidea | <https://ftp.ncbi.nlm.nih.gov/genomes/all/GCF/000/327/385/GCF_000327385.1_Helro1> |

Table S2 Location information for *C. sikamea* populations sampled from China, Japan and the US.

| Population | Location | Abbreviation | Individuals | Latitude | Longitude |
| --- | --- | --- | --- | --- | --- |
| China | Haimen,Nantong | NT | 20 | 32.11 | 121.49 |
|  | Xiangshan Bay,Zhejiang | ZJ | 24 | 29.48 | 121.42 |
|  | Xinglin Bay,Fujian | FJ | 20 | 24.56 | 118.07 |
|  | Yangxi,Guangdong | GD | 19 | 21.65 | 111.78 |
|  | Qinzhou,Guangxi | GX | 20 | 21.67 | 108.65 |
| Japan | Kagami River Estuary, Kumamoto | JP | 20 | 32.6 | 130.60 |
| United States | Commercially cultured (Dabob bay) | US | 18 | 47.22 | -123.04 |

Table S3 Summary statistics of sequencing data used for genome assembly annotation.

| Goal | | Sequencing platform | Clean data | Coverage (×) |
| --- | --- | --- | --- | --- |
| Genome assembly | Genome survey | Novaseq PE150 | 68.08G | 112 |
|  | Genome sequencing | PacBio Sequel II | 815.58G | 1324 |
|  | HIC | Illumina Novaseq PE150 | 69.77G | 114 |
| Genome annotation aiding | Full-length transcriptome | PacBio Sequel II | 51.18G |  |
|  | NGS transcriptome | Illumina Novaseq PE150 | 40.8G |  |

Table S4 K-mer statistics from *C. sikamea* genome survey analysis

| Sample | *Crassostrea sikamea* |
| --- | --- |
| K-mer | 21 |
| K-mer number | 57113044418 |
| K-mer depth | 109 |
| Genome size(Mbp) | 523.97 |
| Repeat(%) | 31.65 |
| Heterozygous rate(%) | 3.38 |

Table S5 Statistics for contig level assembly of *C. sikamea* genome.

| Items | Contig_len(bp) | Contig_num | Scaffold_len(bp) | Scaffold_num |
| --- | --- | --- | --- | --- |
| Total | 616,487,218 | 282 | 616,510,369 | 27 |
| Max | 15,301,709 | - | 74,345,098 | - |
| Number>=2000bp | - | 282 | - | 27 |
| N50 | 4,205,225 | 43 | 62,248,876 | 5 |
| N60 | 3,407,547 | 59 | 61,790,015 | 6 |
| N70 | 2,665,734 | 79 | 60,173,592 | 7 |
| N80 | 1,817,346 | 106 | 58,191,363 | 8 |
| N90 | 1,098,807 | 148 | 54,544,394 | 9 |

Table S6 Statistics for chromosome level assembly of *C. sikamea* genome.

| Pseudomolecule | Length (bp) |
| --- | --- |
| chr1 | 74345098 |
| chr2 | 73426816 |
| chr3 | 66709294 |
| chr4 | 62897016 |
| chr5 | 62248876 |
| chr6 | 61790015 |
| chr7 | 60173592 |
| chr8 | 58191363 |
| chr9 | 54544394 |
| chr10 | 41404708 |
| Total anchored | 615731172 |
| Unanchored | 779197 |

Table S7 Mapping and coverage rate based on whole-genome short sequence reads.

| Sample | *Crassostrea sikamea* |
| --- | --- |
| Clean Reads | 453,899,788 |
| Clean Bases | 68,084,968,200 |
| Mapped Reads | 439,746,189 |
| Mapped Reads Rate (%) | 96.88 |
| Mapped Bases | 64,301,213,929 |
| Mapped Bases Rate (%) | 94.44 |
| Mean Depth | 104.4 |
| Coverage Rate (%) | 99.82 |

Table S8 Repeat content in *C. sikamea* genome predicted with different methods.

| Strategies | Type | Repeat length(bp) | % of genome |
| --- | --- | --- | --- |
| Homologous | RepeatMasker | 214,385,753 | 34.78 |
|  | RepeatProteinMask | 30,900,041 | 5.01 |
| *De novo* | RepeatModeler | 246,171,832 | 39.93 |
|  | TRF | 39,263,188 | 6.37 |
|  | Total | 289,860,694 | 47.02 |

Table S9 Types and proportion of different transposable elements in *C. sikamea* genome.

| Class | RepeatMasker TEs | | RepeatProteinMask TEs | | RepeatModeler TEs | | Combined TEs | |
| --- | --- | --- | --- | --- | --- | --- | --- | --- |
| Type | Length(bp) | % Genome | Length(bp) | % Genome | Length(bp) | % Genome | Length(bp) | % Genome |
| DNA | 107,031,619 | 17.36 | 662,869 | 0.11 | 2,978,448 | 0.48 | 108,599,332 | 17.62 |
| LINE | 12,575,353 | 2.04 | 4,995,644 | 0.81 | 11,905,754 | 1.93 | 17,185,253 | 2.79 |
| SINE | 301,716 | 0.05 | 0 | 0 | 0 | 0 | 301,716 | 0.05 |
| LTR | 12,348,872 | 2.00 | 10,959,102 | 1.78 | 4,692,374 | 0.76 | 18,331,194 | 2.97 |
| Unknown | 3,220,160 | 0.52 | 0 | 0 | 221,471,243 | 35.92 | 221,716,974 | 35.96 |
| Other | 83,226,402 | 13.50 | 14,334,932 | 2.33 | 6,362,459 | 1.03 | 96,880,383 | 15.71 |
| Total | 214,385,753 | 34.78 | 30,900,041 | 5.01 | 246,171,832 | 39.93 | 280,485,679 | 45.50 |

Table S10 Types and proportion of noncoding RNA in *C. sikamea* genome.

| Class | Type | Copy | Average length(bp) | Total length(bp) | % of genome |
| --- | --- | --- | --- | --- | --- |
| miRNA | miRNA | 308 | 176 | 54206 | 0.00879 |
| tRNA | tRNA | 3695 | 75 | 276262 | 0.04481 |
|  | 18S | 33 | 332 | 10962 | 0.00178 |
| rRNA | 28S | 84 | 201 | 16890 | 0.00274 |
|  | 5.8S | 6 | 153 | 918 | 0.00015 |
|  | 5S | 305 | 93 | 28485 | 0.00462 |
|  | CD-box | 20 | 107 | 2148 | 0.00035 |
| snRNA | HACA-box | 21 | 174 | 3654 | 0.00059 |
|  | splicing | 134 | 166 | 22187 | 0.00360 |
| Total |  |  |  | 415712 | 0.06743 |

Table S11 Statistics for gene prediction by different methods

|  | Method | Number of proteins | Average gene length(bp) | Average cds length(bp) | Average exons per gene | Average exon length(bp) | Average intron length(bp) |
| --- | --- | --- | --- | --- | --- | --- | --- |
| *ab initio* | Augustus | 33449 | 8057 | 1443 | 6.94 | 208 | 1115 |
|  | GeneMark | 39137 | 7654 | 1327 | 7.34 | 181 | 999 |
|  | SNAP | 76481 | 9242 | 829 | 6.52 | 127 | 1524 |
| RNA-seq | PASA | 9726 | 8914 | 1278 | 6.37 | 261 | 1349 |
| Homology | *C.gigas* | 39613 | 5582 | 1053 | 5.55 | 190 | 997 |
|  | *C.hongkongensis* | 38941 | 3905 | 798 | 4.12 | 194 | 998 |
|  | *C.virginica* | 34948 | 5499 | 1048 | 5.4 | 194 | 1012 |
|  | *Homo.sapiens* | 12467 | 4052 | 834 | 4.56 | 183 | 906 |
|  | *S.glomerata* | 35516 | 5402 | 984 | 4.94 | 199 | 1123 |
|  | EVM | 37121 | 7289 | 1362 | 6.79 | 201 | 1025 |

Table S12 Statistics for *C. sikamea* genes predicted with different organisms based on homologous genes in related species

| Gene set | Number of proteins | Average gene length(bp) | Average cds length(bp) | Average exons per gene | Average exon length(bp) | Average intron length(bp) |
| --- | --- | --- | --- | --- | --- | --- |
| *C.gigas* | 31371 | 9090 | 1615 | 7.9 | 204 | 1086 |
| *C.virginica* | 34596 | 8968 | 1627 | 8.32 | 196 | 1006 |
| *S.glomerata* | 29738 | 8120 | 1529 | 7.84 | 195 | 965 |
| *Homo sapiens* | 22311 | 43411 | 1615 | 9.29 | 174 | 5042 |
| *C.hongkongensis* | 25675 | 5030 | 1139 | 5.81 | 196 | 810 |

Table S13 Gene homologues annotated in different databases.

| Database | Count | Percentage(%) |
| --- | --- | --- |
| Uniprot-BLASTP | 20903 | 56.31 |
| Uniprot-BLASTX | 20388 | 54.92 |
| GO | 21697 | 58.45 |
| KEGG | 11722 | 31.58 |
| Map | 6762 | 18.22 |
| NR | 34724 | 93.54 |
| NT | 36579 | 98.54 |
| PFAM | 21959 | 59.16 |
| eggNOG | 17051 | 45.93 |
| Total_annotated | 36989 | 99.64 |
| Total_unigene | 37121 | 100.00 |

Table S14 Mapping rate and average depth of re-sequencing data from different populations.

| Population | N | Mapping rate | Average depth |
| --- | --- | --- | --- |
| NT | 20 | 91.65% | 16.79 |
| ZJ | 24 | 91.71% | 16.55 |
| FJ | 20 | 91.69% | 17.19 |
| GD | 19 | 91.72% | 17.25 |
| GX | 20 | 91.79% | 17.06 |
| JP | 20 | 91.17% | 16.28 |
| US | 18 | 91.12% | 16.69 |
| ALL | 141 | 91.56% | 16.82 |

Table S15 Genome-wide SNP annotation and statistics after filtering

| Category | | Number of SNPs |
| --- | --- | --- |
| Upstream | | 104205 |
| Exonic | Stop gain | 933 |
|  | Stop loss | 126 |
|  | Synonymous | 262430 |
|  | Non-synonymous | 102804 |
| Intronic | | 726232 |
| Splicing | | 623 |
| Downstream | | 110841 |
| Upstream/Downstream | | 5978 |
| Intergenic | | 598231 |
| **Total** | | **1954312** |

Note: Upstream means the SNP located in 1 kb upstream region of genes; EXONIC: The mutation is located in the exon region; STOP GAIN: Mutations that cause genes to acquire stop codons; STOP LOSS: A mutation that causes a gene to lose its stop codon; SYNONYMOUS: synonymous variation; NONSYNONYMOUS: Non synonymous variation; INTRONIC: The mutation is located in the intron region; SPLICING: The mutation is located at the splice site (2bp near the exon/intron boundary in introns); Downstream: 1 kb downstream region of the gene; Upstream/Downstream: 1 KB region upstream of a gene, and also 1 kb region downstream of another gene; INTERGENIC: The variation is located in the intergenic region.

Table S16 Population fixation index (Fst) data for seven populations based on genome-wide SNPs.

|  | ZJ | NT | FJ | GD | GX | JP | US |
| --- | --- | --- | --- | --- | --- | --- | --- |
| ZJ | 0 |  |  |  |  |  |  |
| NT | 0.00007 | 0 |  |  |  |  |  |
| FJ | 0.00027 | 0.00003 | 0 |  |  |  |  |
| GD | 0.00018 | 0.00007 | -0.00039 | 0 |  |  |  |
| GX | 0.00200 | 0.00157 | 0.00152 | 0.00117 | 0 |  |  |
| JP | **0.02737** | **0.02725** | **0.02721** | **0.02684** | **0.02659** | **0** |  |
| **US** | **0.05720** | **0.05715** | **0.05718** | **0.05733** | **0.05750** | **0.03295** | **0** |

Table S17 Blocks showing selection sweep signals and associated genes summary from different comparisons.

| Comparison | Blocks | Chromosomes | Gene numbers |
| --- | --- | --- | --- |
| **US vs. JP** | **79** | **8** | **402** |
| **JP vs. ZJ** | **201** | **10** | **768** |
| **US vs. ZJ** | **154** | **10** | **742** |
| **US vs.JP and US vs. ZJ shared** | **22** | **8** | **118** |
| **ZJ vs.JP and ZJ vs.US shared** | **76** | **10** | **356** |
